# Zoledronic Acid Targeting of the Mevalonate Pathway Causes Reduced Cell Recruitment and Attenuates Pulmonary Fibrosis

**DOI:** 10.3389/fphar.2022.899469

**Published:** 2022-06-02

**Authors:** Lloyd Tanner, Jesper Bergwik, Andrew B. Single, Ravi K. V. Bhongir, Jonas S. Erjefält, Arne Egesten

**Affiliations:** ^1^ Respiratory Medicine, Allergology, and Palliative Medicine, Department of Clinical Sciences Lund, Lund University and Skåne University Hospital, Lund, Sweden; ^2^ Unit of Airway Inflammation, Department of Experimental Medical Sciences, Lund University, Lund, Sweden

**Keywords:** IPF, Zoledronic Acid, mevalonate, Fibrosis, FDPS, drug repurposing

## Abstract

**Background and aim:** Idiopathic pulmonary fibrosis (IPF) is a progressive lung disease causing irreparable scarring of lung tissue, with most patients succumbing rapidly after diagnosis. The mevalonate pathway, which is involved in the regulation of cell proliferation, survival, and motility, is targeted by the bisphosphonate zoledronic acid (ZA). The aim of this study was to assess the antifibrotic effects of ZA and to elucidate the mechanisms by which potential IPF treatment occurs.

**Methods:** A series of *in vitro* and *in vivo* models were employed to identify the therapeutic potential of ZA in treating IPF. *In vitro* transwell assays were used to assess the ability of ZA to reduce fibrotic-related immune cell recruitment. Farnesyl diphosphate synthase (FDPS) was screened as a potential antifibrotic target using a bleomycin mouse model. FDPS-targeting siRNA and ZA were administered to mice following the onset of experimentally-induced lung fibrosis. Downstream analyses were conducted on murine lung tissues and lung fluids including 23-plex cytokine array, flow cytometry, histology, Western blotting, immunofluorescent staining, and PCR analysis.

**Results:** *In vitro* administration of ZA reduced myofibroblast transition and blocked NF-κB signaling in macrophages leading to impaired immune cell recruitment in a transwell assay. FDPS-targeting siRNA administration significantly attenuated profibrotic cytokine production and lung damage in a murine lung fibrosis model. Furthermore, ZA treatment of mice with bleomycin-induced lung damage displayed decreased cytokine levels in the BALF, plasma, and lung tissue, resulting in less histologically visible fibrotic scarring. Bleomycin-induced upregulation of the ZA target, FDPS, was reduced in lung tissue and fibroblasts upon ZA treatment. Confirmatory increases in FDPS immunoreactivity was seen in human IPF resected lung samples compared to control tissue indicating potential translational value of the approach. Additionally, ZA polarized macrophages towards a less profibrotic phenotype contributing to decreased IPF pathogenesis.

**Conclusion:** This study highlights ZA as an expedient and efficacious treatment option against IPF in a clinical setting.

## Introduction

Idiopathic pulmonary fibrosis (IPF) is a disorder characterized by progressive lung scarring with a median survival time of 3 years postdiagnosis (Raghu et al., 2011, 2019; Chang et al., 2020). IPF is associated with increasing cough and dyspnea, affecting approximately 3 million people worldwide (Martinez et al., 2017; Lederer and Martinez, 2018). Mechanistically, IPF is thought to be driven by chronic and repeated epithelial injury-repair responses leading to epithelial-mesenchymal transition and other resultant changes (Wynn and Vannella, 2016; Skibba et al., 2020; Baratella et al., 2021). Following repeated epithelial damage, fibroblasts and epithelial cells are triggered to transition, migrate, and proliferate resulting in excessive extracellular matrix (ECM) deposition (Hu and Phan, 2013; Cutolo et al., 2018; Piera-Velazquez and Jimenez, 2018; Zhang et al., 2019; Skibba et al., 2020).

Additionally, inflammatory cells such as monocytes and tissue-resident macrophages play crucial roles in tissue injury and wound healing responses, displaying diverse phenotypes to control a multitude of regulatory functions during IPF progression (Vannella and Wynn, 2017; Upagupta et al., 2018; Zhang et al., 2018; Chakarov et al., 2019; Guilliams et al., 2020; Henderson et al., 2020). Current IPF therapies focus on the inhibition of collagen deposition by blocking myofibroblast activation and proliferation, with limited success in achieving overall IPF resolution, necessitating the need for novel therapies (Lederer and Martinez, 2018). Drug repurposing allows for shortened preclinical and clinical trial periods from an estimated 10–12 years to 3–4 years, allowing patients to benefit from cheaper medications (Xue et al., 2018; Fetro and Scherman, 2020).

In this study we repurposed zoledronic acid (ZA), a drug commonly used for the treatment of post-menopausal onset of osteoporosis and hypercalcemia associated with multiple myeloma (Watts and Diab, 2010), in *in vitro* and *in vivo* models of pulmonary fibrosis. In our study, a bleomycin-challenged murine model was used to assess ZA’s anti-fibrotic efficacy. Bleomycin-induced fibrosis reproduces several phenotypic features of human IPF, including peripheral alveolar septal thickening, dysregulated cytokine production, and immune cell influx (Tashiro et al., 2017; Tanner and Single, 2019).

The primary target of bisphosphonates is farnesyl diphosphate synthase (FDPS), a key enzyme in the mevalonate pathway (Dunford et al., 2001). Isoprenoid intermediates derived from the mevalonate pathway are utilized in post-translational modifications of several proteins, including Rho GTPases, regulating important cellular functions including modulation of the actin cytoskeleton (Tsou et al., 2014; Larson-Casey et al., 2019). Taken together, increased mevalonate pathway intermediates facilitate the differentiation and movement of pro-fibrotic cell-types and an increased production of reactive oxygen species, potentially leading to IPF onset (Zeng et al., 2003; Chen et al., 2007; Larson-Casey et al., 2016).

We show herein, that FDPS is decreased in control patient lung samples versus IPF lung tissue. Subsequent administration of FDPS-targeting siRNA directly to the lung, reduced bleomycin induced fibrotic lung damage. Similarly, bleomycin administered mice displayed increased *FDPS* expression versus ZA-administered animals. In an IPF context, increased profibrotic macrophage populations have been found in the blood of IPF patients as well as in the lungs of bleomycin-treated mice (Zhou et al., 2014). We show that ZA administration confers beneficial macrophage repolarization and modulation of the fibrotic response, highlighting FDPS as a tractable therapeutic target. ZA is particularly interesting in a clinical context due to its FDA approval and acceptable safety profile, with transient flu-like symptoms and less-commonly occurring osteonecrosis of the jaw the main reported side effects (Rosen et al., 2003).

The aim of this study was to assess the antifibrotic effects of ZA and to delineate any antifibrotic mechanisms associated with FDPS-targeting. The repurposing of bisphosphonates for use in pulmonary fibrosis-related disease is a unique concept that is not currently described in the literature. If successfully applied in the clinic, the novel treatment strategies described in this study would decrease the rate of disease progression associated with IPF.

## Materials and Methods

### Study Design

Further details regarding the materials and methods used can be found in the Supplementary Material. *In vivo* murine studies using intratracheally-administered bleomycin were chosen as well-established and relevant models of experimental lung fibrosis (Tashiro et al., 2017; Tanner and Single, 2019). Sample sizes were calculated by power analysis based on previous experience, feasibility, and to conform to the ARRIVE guidelines (arriveguidelines.org). For lung fibrosis experiments testing, *n* ≥ 8 to 15 mice per group were used to achieve statistical significance as calculated using G Power Software (ANOVA, with fixed effects, omnibus, one-way). For siRNA experiments, demonstrating FDPS as a target for IPF treatment, *n* = 5 mice per group were used. Mice were randomly assigned to treatment groups. Downstream analyses were conducted with the investigator blinded to the treatment groups, and no animals were excluded as outliers from the reported dataset. All *in vitro* and *in vivo* experiments were performed in 2–4 technical replicates. Macroscopically normal, tumor-free lung tissue samples (control tissue) were obtained during transplantation or resection from patients undergoing cancer surgery or from patients with IPF diagnosed by techniques supplemented by spirometry (Supplementary Table S2). Patients undergoing lung biopsies with confirmed distal lung fibrosis (by histological analysis), or high-resolution computerized tomography (HRCT) scans with characteristic IPF features (e.g., opaque fibrosis-like areas and ‘honeycombing’ in the lung parenchyma) were included in this study (*n =* 4 per group).

### Ethical Approval

All animal experiments in this study were approved by the Malmö-Lund Animal Care Ethics Committee (M17009-18). Lung tissue used for immunoreactivity assays was obtained after written informed consent, approval by the Regional Ethical Review Board in Lund (approval no. LU412-03), and performed in accordance with the Declaration of Helsinki as well as relevant guidelines and regulations.

### Fibroblast Transwell Experiment

Human lung fibroblasts (HFL-1; ATCC, Manassas, VA, United States) were cultured until confluent in complete growth medium supplemented with l-glutamine, 100 U/mL penicillin, 100 μg/ml streptomycin, and 10% FBS. Fibroblast chemotaxis was measured using 24-well Nunc (8-µm pore size) Transwell inserts (Thermo Fisher, Waltham, MA, United States). Following confluence, cells were seeded (5 × 10^5^ cells/ml) into the upper chamber in FBS-free medium, whilst the lower chamber contained complete medium with additional 10% FBS as a chemoattractant. Experiments were carried out in experimental triplicate, with each experiment containing 4 biological replicates. Medium containing transforming growth factor beta 1 (TGF-β1; 2 ng/ml) followed by the addition of medium containing zoledronic acid (ZA; 2 μM), medium only, or vehicle only (dimethyl sulfoxide; DMSO 2% and phosphate-buffered saline (PBS) pH 7.4). Following 48 h, medium was removed and cells in the lower chamber were stained (crystal violet) and imaged using a Nikon Eclipse 80i Compound Fluorescent Microscope and analyzed using Nikon NIS Elements F4.60.00.

### MTT Assay

Mosmann’s MTT [3-(4,5-dimethylthiazol-2-yl)-2,5-diphenyltetrazolium bromide] assay was used to determine cell viability in both RAW264.7 and THP-1 cells. After the addition of the compounds at a starting concentration of 1000 μM (serial diluted to 0.1 μM), and incubation for 48 h, each well received MTT at a concentration of 5 mg/ml in phosphate-buffered saline (PBS), with blank samples receiving only medium and MTT. Each compound concentration tested in this study was completed in triplicate. DMSO was added to each well, followed by plate shaking for 5 min to dissolve the formazan crystals. The absorbance of the formazan salt was measured at 540 nm by a VICTOR 1420 Multilabel plate reader. The following formula Eq. 1 was used to calculate the cell viability:

Percentage viability=sample absorbance − blank absorbance/control absorbance − blank absorbance X100 (1)

Nonlinear dose response curves were constructed using GraphPad Prism 4 software (GraphPad Software, San Diego, CA, United States) and Microsoft Excel.

### Immunostaining of Embryonic Mouse Fibroblasts

Murine C57BL/6 embryonic fibroblasts (MEFs) were seeded (1 × 10^4^ cells/mL) into 24-well plates containing rounded glass cover slips. TGF-β1 (2 ng/ml) was added to each well, followed by the addition of medium containing ZA or vehicle only. Following 48 h of treatment, cells were washed and fixed with ice cold methanol, containing 0.5% Triton-X100. Slides were blocked using Dako Protein Block (Agilent, Santa Clara, CA, United States) for 1 h at room temperature and then stained with primary antibodies (overnight), rabbit anti-COL1AI, rabbit anti-αSMA, and rabbit anti-fibronectin (Abcam, Cambridge, United Kingdom). Alexa Fluor 488-conjugated goat anti-rabbit antibody (Invitrogen, Carlsbad, CA, United States) was used as secondary antibody (2 h). Glass cover slips were removed and mounted onto glass slides, with nuclei counterstained using DAPI containing fluoroshield (Abcam). Images were visualized using a Nikon Eclipse 80i Compound Fluorescent Microscope and analyzed using Nikon NIS Elements F4.60.00.

### *Ex vivo* Cell Recruitment Assay

BAL was performed on C57BL/6 mice (*n* = 10) with a total volume of 1 ml PBS containing 100 μM EDTA. BALF was collected in Eppendorf tubes on ice and immediately separated using the Anti-F4/80 MicroBeads UltraPure (mouse; 130-110-443), dead cell removal kit (130-090-101), MS columns (130-042-201), and QuadroMACS™ Separator (130-090-976; Miltenyi Biotec, Bergisch Gladbach, Germany). Purified murine macrophages were counted and run on a BD Accuri C6 flow cytometer to determine cell purity. Concurrently, murine blood was isolated *via* heart puncture and collected in EDTA-containing tubes. Murine PBMCs were isolated using Ficoll-Paque PREMIUM 1.084 (Sigma-Aldrich, Saint Louis, MO, United States) density centrifugation (400 x *g* for 40 min) and subsequently counted. In the transwell plate, murine macrophages were added to the bottom well (5 × 10^5^ cells/well), with murine PBMCs (6 × 10^6^ cells/well) added to the top chamber. Following 24 h of RANKL (10 ng/ml) macrophage stimulation, cells were harvested and stained for flow cytometry analysis carried out using a BD Accuri C6 (BD, Franklin Lakes, NJ, United States). Samples were separated into 3 aliquots, 1) alveolar macrophages as identified by F4/80 (BM8; 11-4321-42), CD11b (BD553312), and I-A/I-E (BD557000); 2) B and T cells as identified by CD45 (BD553079), CD3 Molecular Complex (BD 555276), CD19 (BD561736); 3) macrophage subtypes and neutrophils as identified by CD11b (BD553312), CD11c (BD558079), Ly-6G (BD551461).

### Mouse Macrophage (M1/M2) *Ex Vivo* Assay

BAL was performed on C57BL/6 mice (*n* = 10) with a total volume of 1 ml PBS containing 100 μM EDTA. BALF was collected in Eppendorf tubes on ice and immediately separated using the Anti-F4/80 MicroBeads UltraPure and QuadroMACS™ Separator as indicated above. Isolated murine macrophages were added to 12-well plates at 5 × 10^8^ cells/well. Cells were stimulated with interleukin-4 (IL-4) and IL-13 (20 ng/ml, PeproTech, NJ, United States) for 48 h followed by flow cytometry analysis (BD Accuri C6) to assess M1/M2 phenotype. Cells were gated using the following antibodies: CD206 (BioLegend, 141705), CD11b (BD553312), CD11c (BD558079), and F4/80 (BD 565635).

### Animals

10-12-week-old male C57BL/6 mice (Janvier, Le Genest-Saint-Isle, France) were housed at least 2 weeks in the animal facility at the Biomedical Service Division at Lund University before initiating experiments and were provided with food and water *ad libitum* throughout the study. Mice were randomly allocated into five groups (*n* = 4-15 animals per group): intratracheally (i.t.)-administered bleomycin (2.5 U/kg) + vehicle (i.p./i.t.), bleomycin (i.t.) +ZA (0.1 mg/kg; i. p./i.t.), saline (i.t.) + vehicle (i.p./i.t.), and saline (i.t.) + ZA (0.1 mg/kg; i. p./i.t.).

### siRNA Administration

For lung fibrosis experiments testing the viability of *Fdps* as a drug target in siRNA experiments, *n* ≥ 4 to 5 mice per group were used to achieve statistical significance. Mice were randomly assigned to treatment groups. For the siRNA experiments, lung fibrosis was induced by intratracheal introduction of bleomycin (2.5 U/kg) or saline as control. Following 14 days, siRNA targeting mouse *Fdps* (L-059452-01–0020**)** or nontargeting control (D-001810-01-50) mRNA (Horizon Discovery, Water Beach, United Kingdom) was administered intratracheally at either 25 μg per mouse (*Fdps* siRNA) or 25 μg of nontargeting siRNA. On day 21, mice were euthanized followed by collection of blood, lung tissue, and BALF. Downstream analyses were conducted with the investigator blinded to the treatment groups, and no animals were excluded as outliers from the reported dataset. All *in vitro* and *in vivo* experiments were performed in two to four technical replicates. Human resected lung sections were obtained with informed consent, with a statistically significant *n =* 4 samples used for disease and control samples.

### Blood Collection

Blood was collected in 0.5 M EDTA tubes by cardiac puncture and centrifuged at 1,000 x *g* for 10 min. Plasma supernatant was used for the analysis of inflammatory mediators using a multiplex assay (Bio-plex assay; Bio-Rad, Hercules, CA, United States).

### Collection of Lung Tissue

Right lungs were collected in Eppendorf tubes on dry ice and stored at -80°C. The snap-frozen lungs were thawed and homogenized in tissue protein extraction reagent (T-PER) solution (Thermo Fisher Scientific) containing protease inhibitor (Pefabloc SC; Sigma-Aldrich) at a final concentration of 1 mM. Lung homogenates were centrifuged at 9,000 x *g* for 10 min at 4°C, and the supernatants were collected for multiplex analysis. Left lungs were collected in Histofix (Histolab, Göteborg, Sweden) and submerged in 4% buffered paraformaldehyde solution.

### Bronchoalveolar Lavage Fluid (BALF) Collection

BAL was performed with a total volume of 1 ml PBS containing 100 μM EDTA. BALF was collected in Eppendorf tubes on ice, with aliquots made for flow cytometry, cytospin differential counts, and an aliquot transferred to -80°C for multiplex cytokine analysis. Cytospin preparations of cells were stained with modified Wright-Giemsa stain (Sigma-Aldrich).

### SDS-PAGE and Western Blotting

Lung homogenate lysates were analyzed for total protein concentration (Pierce BCA Protein Assay Kit, Thermoscientific). Samples were run on Mini-PROTEAN^®^ Precast Mini polyacrylamide gel electrophoresis (PAGE) Gels (BioRad). Samples were transferred to Trans-Blot Turbo Mini 0.2 µm; polyvinylidene difluoride (PVDF) Transfer Packs with blots incubated overnight with primary antibodies (1:1000) in blocking buffer, collagen 1A1 (COL1A1; ab88147), arginase 1 (ARG1; ab239731), cluster of differentiation 206 (CD206; ab64693), glyceraldehyde 3-phosphate dehydrogenase (GAPDH; ab8245), farnesyl diphosphate synthase (FDPS; ab153805, Abcam), alpha smooth muscle actin (α-SMA; 19245**,** cell signaling). Blots were washed with PBS-Tween, before 1 h incubation with Alexa Fluor 488-conjugated goat anti-rabbit/mouse secondary antibodies (Invitrogen, Carlsbad, CA, United States). Blots were imaged using a BioRad ChemiDoc system. Quantification was performed *via* densitometry and normalized using GAPDH.

### Flow Cytometry

Flow cytometry was carried out using a BD Accuri C6 (BD). The washed cells were incubated with Fixable Viability Stain 510 (FVS510) (BD564406) to differentiate live and dead cells. Cells were washed with Stain buffer 1x (BD554656) and incubated with Lyse Fix 1x (BD558049 (5x)). Fixed cells were washed with stain buffer and aliquoted into two parts. One was incubated with CD11b (BD553312), CD11c (BD558079), Ly-6G (BD551461) antibodies and the other aliquot was incubated with CD11c, MHC (BD558593), SiglecF (BD562680) antibodies.

### Bioplex Cytokine Analysis

For the detection of multiple cytokines in BALF, plasma, and lung homogenate, the Bio-Plex Pro mouse cytokine assay (23-Plex Group I; Bio-Rad) was used on a Luminex-xMAP/Bio-Plex 200 System with Bio-Plex Manager 6.2 software (Bio-Rad). A cytometric magnetic bead-based assay was used to measure cytokine levels, according to the manufacturer’s instructions. The detection limits were as follows: Eotaxin (21372.02–1.15 pg/ml), GCSF (124018.4–6.97 pg/ml), GMCSF (1161.99–3.73), IFN-γ (14994.64–0.72 pg/ml), IL-1α (10337.5–0.63 pg/ml), IL-1β (28913.54–1.57 pg/ml), IL-2 (22304.34–1.21 pg/ml), IL-3 (7639.21–0.47 pg/ml), IL-4 (6334.86–0.36 pg/ml), IL-5 (12950.39–0.76 pg/ml), IL-6 (11370.16–0.66 pg/ml), IL-9 (2580.93–2.46 pg/ml), IL-10 (76949.87–4.09 pg/ml), IL-12p40 (323094.58–17.38 pg/ml), IL-12p70 (79308.46–19.51 pg/ml), IL-13 (257172.3–53.85 pg/ml), IL-17 (8355.61–0.5 pg/ml), KC (23377.88–1.3 pg/ml), MCP-1 (223776.6–45.04 pg/ml), MIP-1α (14038.07–0.58 pg/ml), MIP-1β (928.18–2.39 pg/ml), RANTES (4721.74–4.42 pg/ml), and TNF-α (73020.1–4.61 pg/ml). Cytokine measurements for samples were corrected for protein concentration, measured using a Pierce™ BCA Protein Assay Kit (Thermo Fischer Scientific).

### Hydroxyproline Assay

Hydroxyproline levels were determined using the QuickZyme Hydroxyproline Assay kit (Quickzyme Biosciences, Leiden, Netherlands). Hydroxylation of proline groups within the collagen molecule result in the formation of hydroxyproline, with the modified amino acid stabilizing the helical structure in mature collagen (Berg and Prockop, 1973). Hydroxyproline is highly specific for collagen, allowing hydroxyproline measurements to accurately reflect the amount of collagen in fibrotic lung tissue (de Jong et al., 2012). Lung tissues were homogenized as described above. Homogenates were diluted (1:1 vol:vol) with 12 N HCl and hydrolyzed at 95°C for 20 h. After centrifugation at 13,000 × *g* for 10 min, 200 μl from the supernatant was taken and diluted 1:2 with 4 N HCl. Hydroxyproline standard (6.25–300 μM) was prepared in 4 N HCl and transferred to the microtiter plate. Following addition of a chloramine T-containing assay buffer, samples were oxidized for 20 min at RT. Detection reagent containing p-dimethylaminobenzaldehyde was added and after incubation at 60°C for 1 h, absorbance was read at 570 nm with a VICTOR 1420 Multilabel plate reader (PerkinElmer, Waltham, MA, United States). The hydroxyproline content in lung tissue is given as hydroxyproline (μg) per mg lung tissue, corrected using a Pierce™ BCA Protein Assay Kit (Thermo Fisher Scientific).

### H&E and Picrosirius Red Staining of Lung Tissue

A segment of the left lung was fixed in Histofix (Histolab) and embedded into paraffin and sectioned (3 µm) with a microtome. The tissue sections were placed on slides (Superfrost Plus; Fisher Scientific) and deparaffinized in serial baths of xylene and ethanol followed by staining using Mayer hematoxylin and 0.2% eosin (Histolab) or picrosirius red staining kit (Abcam, Cambridge, United Kingdom). The stained slides were imaged using an Olympus BX60F microscope with an SC50 camera.

### Immunostaining of Murine Lung Sections

Lung tissue sections were fixed as reported above. Lung samples underwent antigen retrieval (pH 9 buffer) using a Dako PT Link pre-treatment module (Agilent). Samples were washed and blocked for 10 min (Dako protein block; Agilent, Santa Clara, CA) before being treated with primary antibodies overnight. Mouse anti-COL1A1, ARG1, and rabbit anti-FDPS, CD206 (Abcam, Cambridge, United Kingdom) antibodies were used. Alexa Fluor 488-conjugated goat/anti-mouse and Alexa Fluor 647 goat/anti-rabbit (Invitrogen, Waltham, CA, United States) were used as secondary antibodies. Glass cover slips were placed onto slides and mounted with DAPI-containing fluoroshield (Abcam). Microscopy was performed on a Widefield Epifluorescence Ti2 microscope equipped with a Nikon DS-Qi2 camera and fluorescence was quantified using ImageJ software.

### Real-Time PCR

Extraction of total mRNA from frozen lung tissue and MEF cells was performed using RNeasy Mini Kit (Qiagen, Valencia, CA, United States), in accordance with manufacturer’s instructions. RNA concentrations were determined using a NanoDrop ND1000 (Saveen Werner AB, Malmö, Sweden). RNA to cDNA conversion was achieved using an iScript Advanced cDNA Synthesis Kit (Bio-Rad). Gene expression was measured using TaqMan™ Fast Advanced Master Mix together with TaqMan™ probes listed in Supplementary Table S1. RT-PCR reactions were analyzed using a QuantStudio™ 7 Flex system (Thermo Fisher Scientific) in 384-well plates. Data was analyzed with QuantStudio™ Real-Time PCR Software v1.3. The obtained Ct-values were normalized to succinate dehydrogenase complex subunit A (*Sdha*) expression, generating ΔCt-values. ΔΔCt-values were obtained by normalizing values to ΔCt-of the PBS only group.

### Immunohistochemistry of Human Lung Samples

Macroscopically normal, tumor-free lung tissue samples were obtained during transplantation or resection from patients undergoing cancer surgery, with IPF patients diagnosed/identified by techniques supplemented by spirometry. Patients undergoing lung biopsies with confirmed distal lung fibrosis (by histological analysis), or high-resolution computerized tomography (HRCT) scans with characteristic IPF features (e.g., opaque fibrosis-like areas and “honeycombing” in the lung parenchyma) were included in this study, with clinical data reported in Supplementary Table S2. Immediately after collection, samples were placed in 4% buffered formaldehyde. Following dehydration and embedding in paraffin, sections (3 µm) were produced. A single staining protocol (EnVision™ Detection system, K5007, Dako, Glostrup, Denmark) was used for visualization of FDPS. Briefly, after antigen retrieval, FDPS was detected using rabbit anti-FDPS (Abcam) antibody (1:1000) and visualized using secondary goat anti-rabbit antibodies conjugated with peroxidase polymers (Dako). Sections were counter-stained with Mayer’s hematoxylin for visualization of background tissue, dehydrated in alcohol/xylene, and mounted on Pertex (Histolab, Göteborg, Sweden). The stained slides were imaged using an Aperio CS2 image capture device. Positive staining was quantified manually (number of cells/area of tissue) or as positivity (positive brown pixels divided by all stained pixels) using computerized image analysis on blinded sections using ImageScope (Aperio, Leica Biosystems, Wetzlar, Germany).

### Statistical Analysis

To assess the effects of ZA in murine models, results were compared using one-way analysis of variance (ANOVA) with Dunnett’s post hoc test. In experiments using two groups, results were compared using an unpaired *t-*test with Welch’s correction. Results in this study are displayed throughout as mean ± standard error of the mean (SEM). Statistical testing was carried out using GraphPad Prism 9.1.1 with statistical significance defined as *p* < 0.05.

## Results

### Zoledronic Acid Reduces Fibroblast to Myofibroblast Transition and Production of Fibrotic Proteins

**FIGURE 1:**
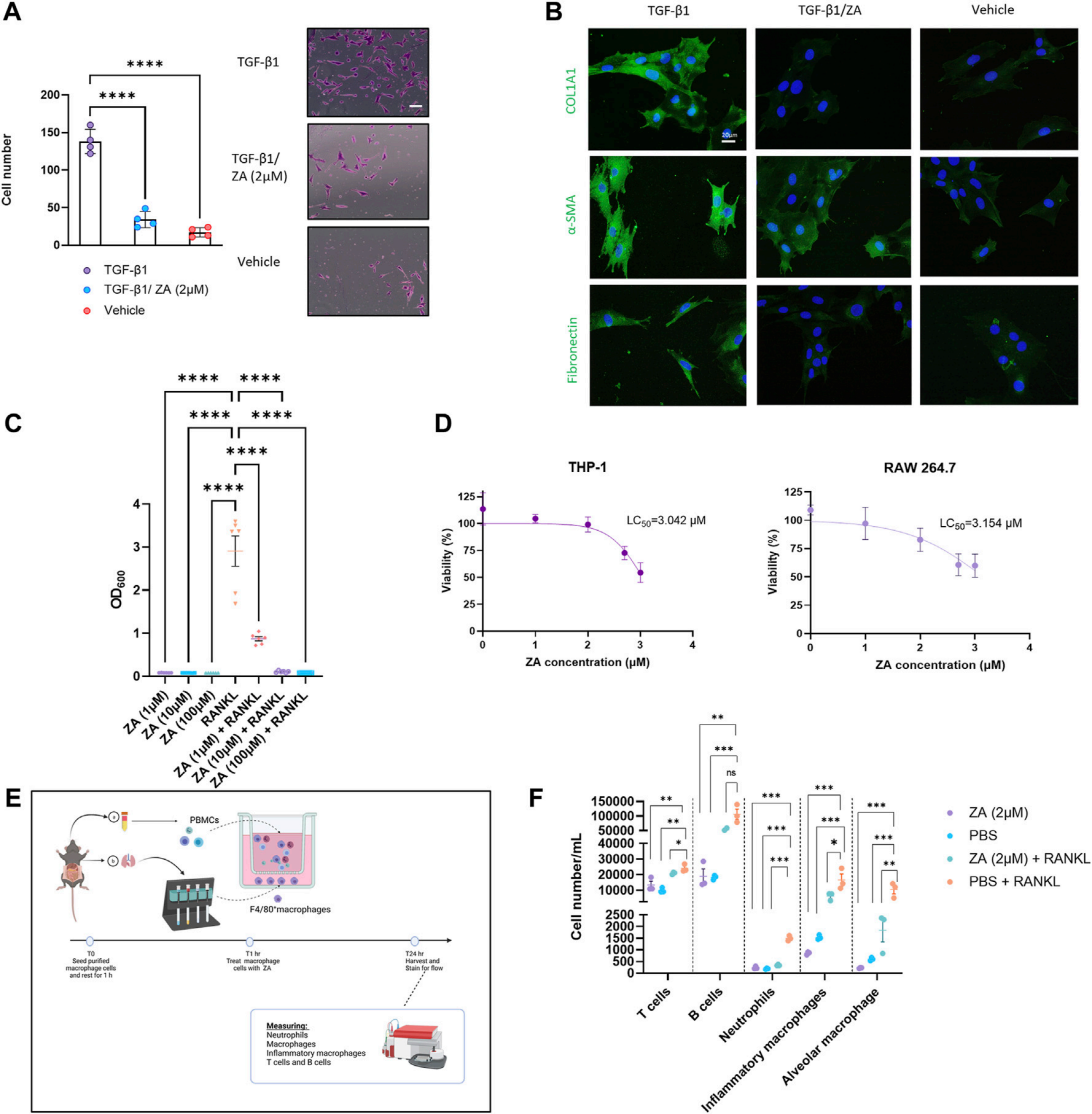
Zoledronic acid (ZA) inhibits *in vitro* transwell migration and fibrotic marker expression, whilst simultaneously reducing nuclear factor kappa-light-chain-enhancer of activated B cells (NF-κB) signaling and immune cell recruitment. **(A)** ZA reduced myofibroblast migration and proliferation in a transwell assay compared to vehicle treatment and no treatment controls (Student’s *t*-test, *p < 0.005* (***) and *p < 0.0001* (****), with representative myofibroblast cells in lower chamber of transwell insert following 48 h shown. **(B)** Immunostaining of fibroblast cells following 48 h of TGF-β1 treatment, with ZA treatment displaying visually reduced levels of collagen, fibronectin and alpha smooth muscle actin (α-SMA) compared to no treatment control. **(C)** RAW264.7 and THP1-Blue™ NF-κB cells were subjected to receptor activator of nuclear factor kappa beta (RANKL) treatment for 48h, with NF-κB signaling significantly reduced by ZA (*p* < 0.0001) and **(D)** low levels of cytotoxicity reported for ZA against both THP-1 and RAW 264.7 cells. **(E,F)** Isolated murine macrophage cells (bottom well) and peripheral blood mononuclear cells (PBMCs) (top well) were exposed to RANKL in a transwell system, with significant reductions in T cells, neutrophils, and macrophage cells following ZA treatment (drug-treated samples were compared to untreated control samples using a one-way ANOVA followed by a Dunnett’s post-hoc test; *N* = 3 technical replicates, from 3 biological replicates per measurement).

To determine ZA involvement in fibroblast to myofibroblast transition (FMT), human lung fibroblasts (HFL-1) were utilized in a transwell assay (Figure 1A). Following stimulation with TGF-β1 for 48 h, cell transition through the transwell membrane was measured. ZA treatment (2 μM) significantly reduced myofibroblast transition (*p* < 0.001). Additionally, staining of mouse embryonic fibroblast cells (MEF) treated with ZA revealed the production of less collagen, fibronectin, and α-smooth muscle actin than in the TGF-β1 control, as measured by immunofluorescence (Figure 1B).

### Reduced NF-κB Macrophage Signaling Moderates Immune Cell Recruitment

To demonstrate the role of ZA in reducing NF-κB signaling, monocytic (THP1-Blue™ NF-κB) cells were stimulated with RANK ligand (RANKL) for 48 h (Figure 1C). ZA significantly reduced RANKL-induced NF-κB signaling at all tested concentrations with ZA not displaying cytotoxicity in either murine macrophages (RAW264.7) or THP1 cells at levels greater than 3 μM (Figure 1D). Following reductions in NF-κB, we examined macrophage-mediated immune cell recruitment from murine peripheral blood inhibition by ZA treatment (Figure 1E). A wide variety of immune cells were measured using flow cytometry and revealed significant decreases between the RANKL and ZA/RANKL treated samples in recruitment of T cells, neutrophils, inflammatory macrophages, and alveolar macrophages (Figure 1F).

### Zoledronic Acid Attenuates Murine Weight Loss and Fibrotic Lung Damage

**FIGURE 2:**
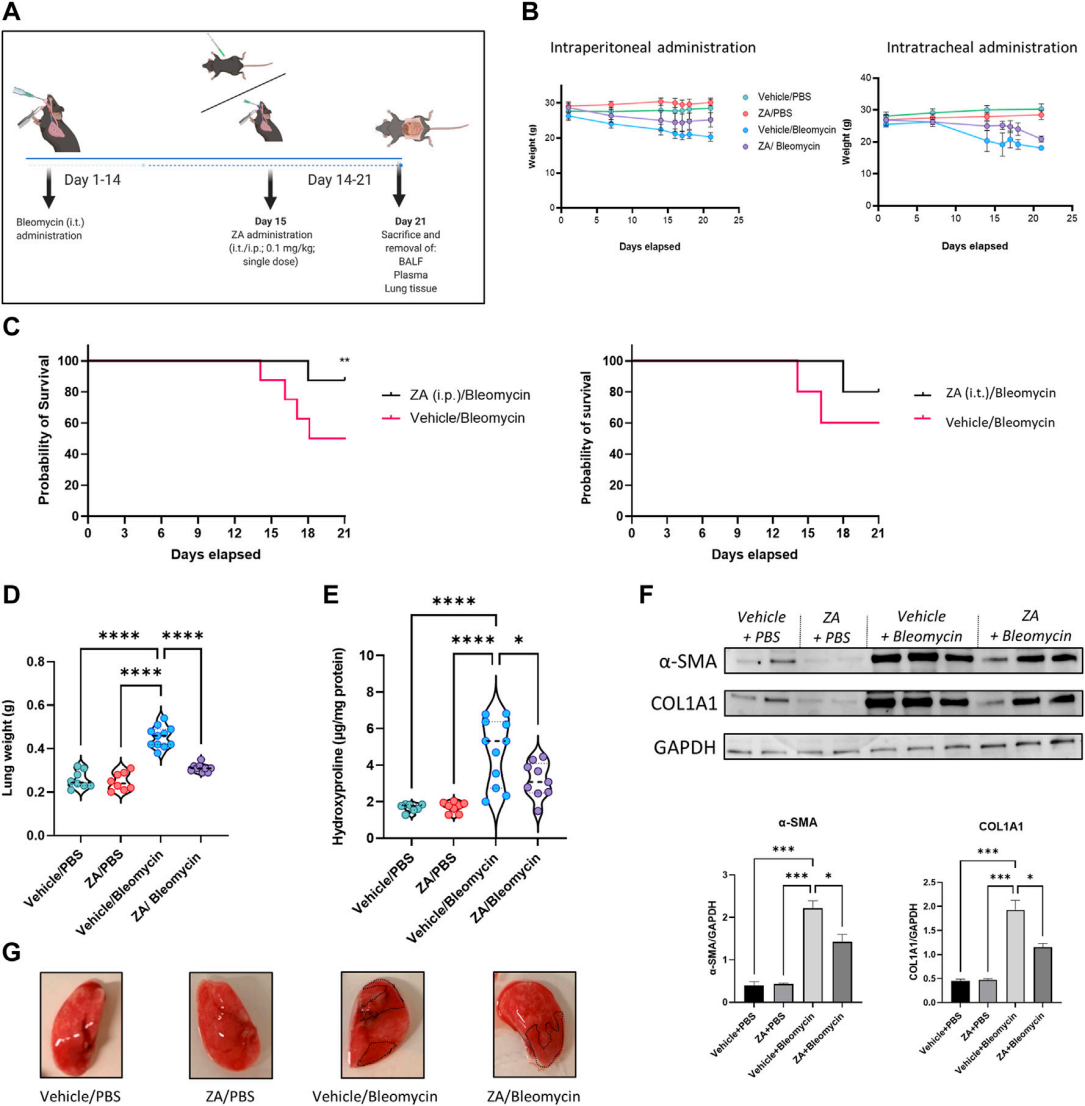
ZA administration, murine weights, survival curve, and lung readouts. **(A)** ZA was administered intraperitoneally (i.p.) and intratracheally (i.t). on day 15 post-bleomycin introduction, with **(B)** murine total weight remaining stable in the ZA (i.p.) group for the duration of 21 days, with both bleomycin groups displaying significant weight loss after 14 days. **(C)** Resultant weight loss in the bleomycin/vehicle group caused significant mortality, with mice in the ZA/Bleo (i.p. and i. t.) groups seemingly protected. **(D)** Murine lung weight showed significant reductions in the ZA treated mice compared to the vehicle/bleomycin group (drug-treated samples were compared to untreated control samples using a one-way ANOVA followed by a Dunnett’s post-hoc test: ****p < 0.0001). **(E)** Hydroxyproline collagen readout displayed significant reduction following ZA (i.p.) treatment (drug-treated samples were compared to untreated control samples using a one-way ANOVA followed by a Dunnett’s post-hoc test: ****p < 0.0001). **(F)** Immunoblotting of ZA-treated murine lung lysates displayed significant reductions in both α-SMA and COL1A1 compared to bleomycin only control lysates **(G)** Representative murine images show bleomycin induced lung damage (collagenous deposition highlighted in dotted areas). Mice were allocated into groups as follows: Vehicle (i.p.)/phosphate buffered saline (PBS; i. t.); zoledronic acid (ZA; i. p.)/PBS (i.t.); Vehicle (i.p.)/Bleomycin (i.t.); ZA (i.p.)/Bleomycin (i.t.).

Given the *in vitro* effect of ZA treatment in reducing fibrotic-related damage, a murine model of bleomycin-induced lung damage was employed, with ZA dosed either intratracheally (i.t.) or intraperitoneally (i.p.; 0.1 mg/kg) at day 15 (Figure 2A). Murine weight loss was dramatically mitigated following i. p. ZA treatment, whilst i. t. ZA treatment offered less protection (Figure 2B). The probability of survival was significantly increased following ZA i. p. administration (*p =* 0.0329), whereas i. t. administration showed no statistically significant difference (Figure 2C). Murine lung weight and hydroxyproline levels provide proxies for generalized lung damage and collagen content, with i. p. administration of ZA reducing both lung weight (*p* < 0.0001) and hydroxyproline levels (*p* = 0.0143) compared to vehicle/bleomycin mice (Figures 2D,E). Additionally, western blot analysis on lung tissue showed a significantly reduced production of the fibrotic proteins COL1A1 and α-SMA after administration of ZA (Figure 2F and Supplementary Figure S8). Images of murine lungs revealed decreased areas of fibrosis after i. p. treatment with ZA (Figure 2G).

### Murine Immune Cell Recruitment to the Lung Is Impaired by Zoledronic Acid

**FIGURE 3:**
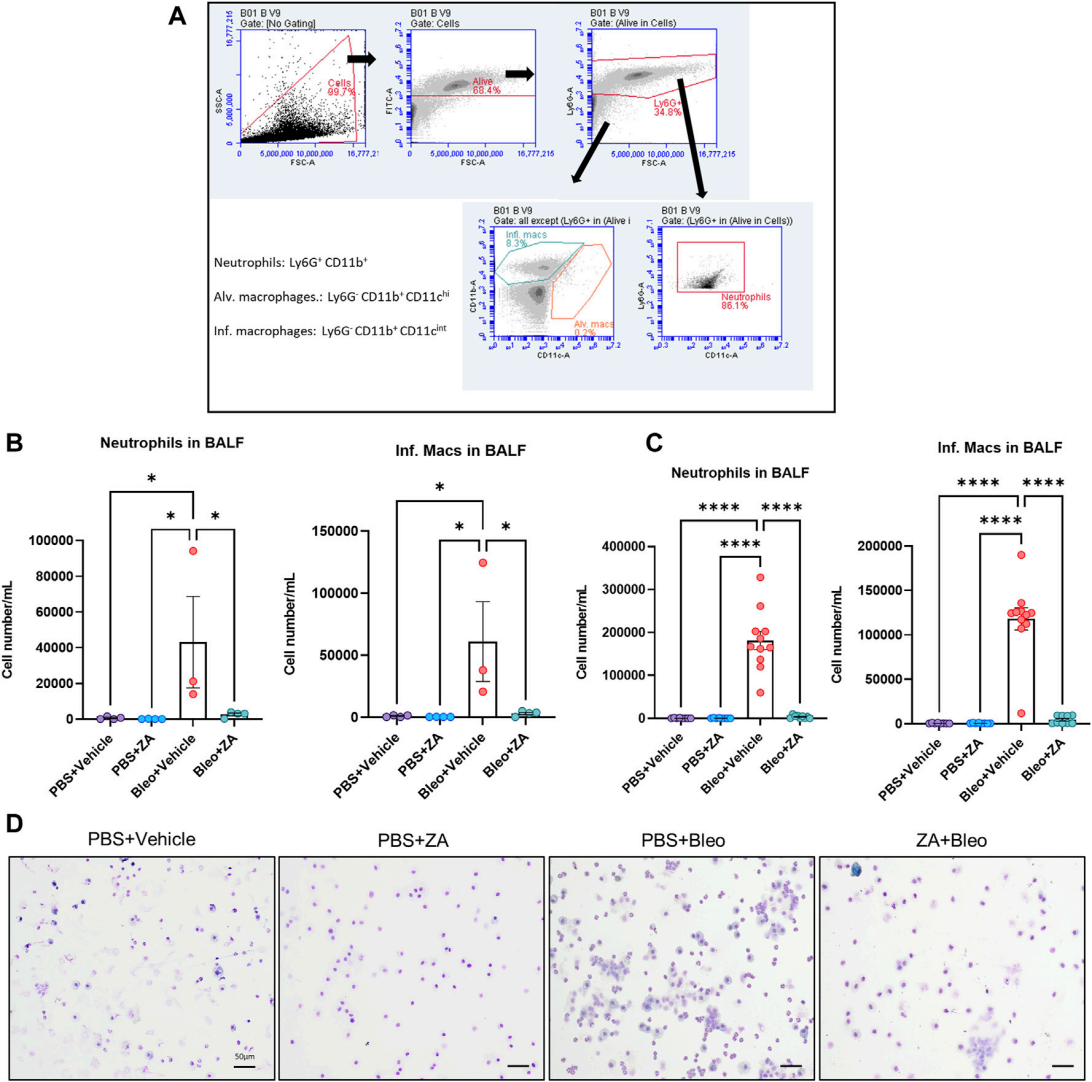
Inflammatory cell influx measured in murine bronchoalveolar lavage fluid (BALF). Murine BALF was assessed for neutrophils and inflammatory macrophages **(B,C)** using flow cytometry, with the representative gating strategy depicted inset **(A)**. Decreased numbers of neutrophils and inflammatory macrophages were detected in response to ZA (i.t.) treatment, with increased significant differences reported between the neutrophils and inflammatory macrophages of mice treated with ZA (i.p.). No significant differences were reported for alveolar macrophage numbers (not shown). Inflammatory cell numbers were compared to the vehicle/bleomycin group using a one-way ANOVA followed by a Dunnett’s post-hoc test (*p < 0.05; ****p < 0.0001). **(D)** Giemsa-Wright stained cytospin slides showing PBS/bleomycin BALF samples containing enlarged inflammatory macrophages, with ZA (i.p.) treatment reducing the presence of inflammatory macrophages. Scale bar = 20 μm.

Administration of ZA effectively reduces regulatory T cell involvement in immune cell recruitment in cancer and fibrosis (Ichikawa et al., 2019; Liu et al., 2019), with a large proportion of these recruited cells being macrophages and neutrophils. Investigation of ZA treatment effects on immune cell infiltration into the airways was performed by flow cytometry on BALF obtained from *in vivo* bleomycin studies at day 21. Decreases in neutrophil count and inflammatory macrophages were seen following both i. p. (*p* < 0.0001) and i. t. (neutrophils: *p* = 0.0417; inflammatory macrophages: *p* = 0.0213) ZA administration compared to vehicle/bleomycin samples (Figures 3A–C). Giemsa-Wright-stained BALF samples showed bleomycin-treated macrophages were enlarged and present in greater number, a phenomenon not seen in mice treated with ZA (i.p.; Figure 3D).

### Murine Lung Structure Is Maintained Following Intraperitoneal ZA Administration

**FIGURE 4:**
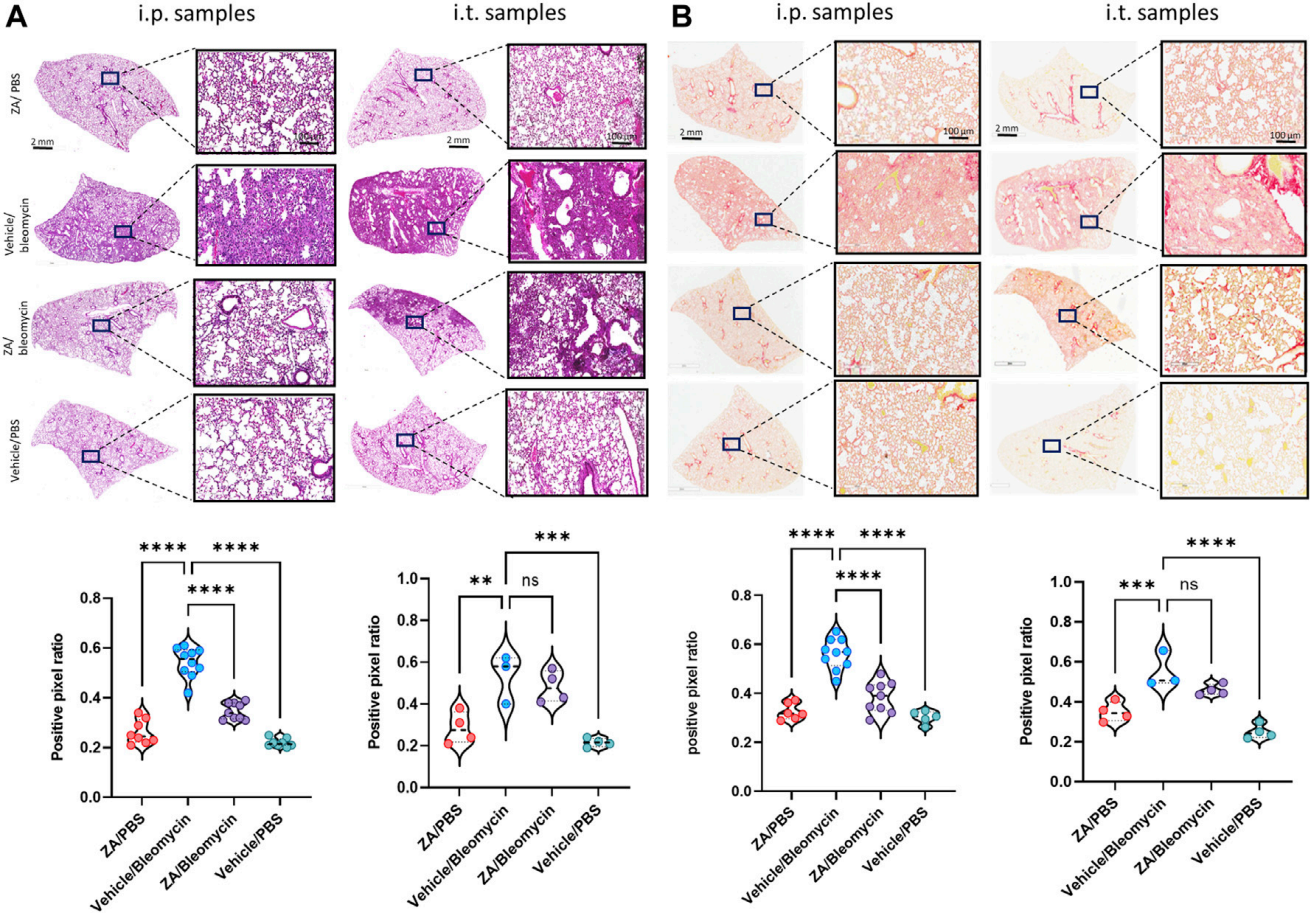
Murine lung staining displays reduced levels of fibrotic-related lung damage following ZA (i.p.) treatment compared to bleomycin/vehicle samples **(A)** ZA (i.p.) significantly decreased lung damage in bleomycin-treated mice (H&E) and **(B)** collagen deposition (picrosirius red) in both macroscopic and microscopic structures compared to vehicle/bleomycin lungs and was confirmed by positive pixel analysis of whole-lung scanned images (scale bar of microscopic image = 100 μm; scale bar of whole lung scan = 2 mm). Statistical analyses were conducted using a one-way ANOVA followed by a Dunnett’s post-hoc test (**p < 0.01; ***p < 0.005; *p < 0.0001).

To determine whether murine histological damage was reduced more efficaciously by a single route of administration, both i. p. and i. t. lung samples from mice treated with ZA were assessed using H&E and picrosirius red staining (Figures 4A,B; Supplementary Figure S1 and 2). ZA administered i. p. quantitatively reduced bleomycin-induced closure of the alveolar structures (*p* < 0.0001), with significant decreases in collagen deposition (*p* < 0.0001) seen in these samples compared to the vehicle/bleomycin samples. Conversely, intratracheally-administered ZA did not confer protection from lung damage as seen in both the H&E (*p* = 0.7009) and picrosirius red staining (*p* = 0.0839).

### Cytokine Production Elucidates Decreased Immune Cell Recruitment Following ZA Treatment

**FIGURE 5:**
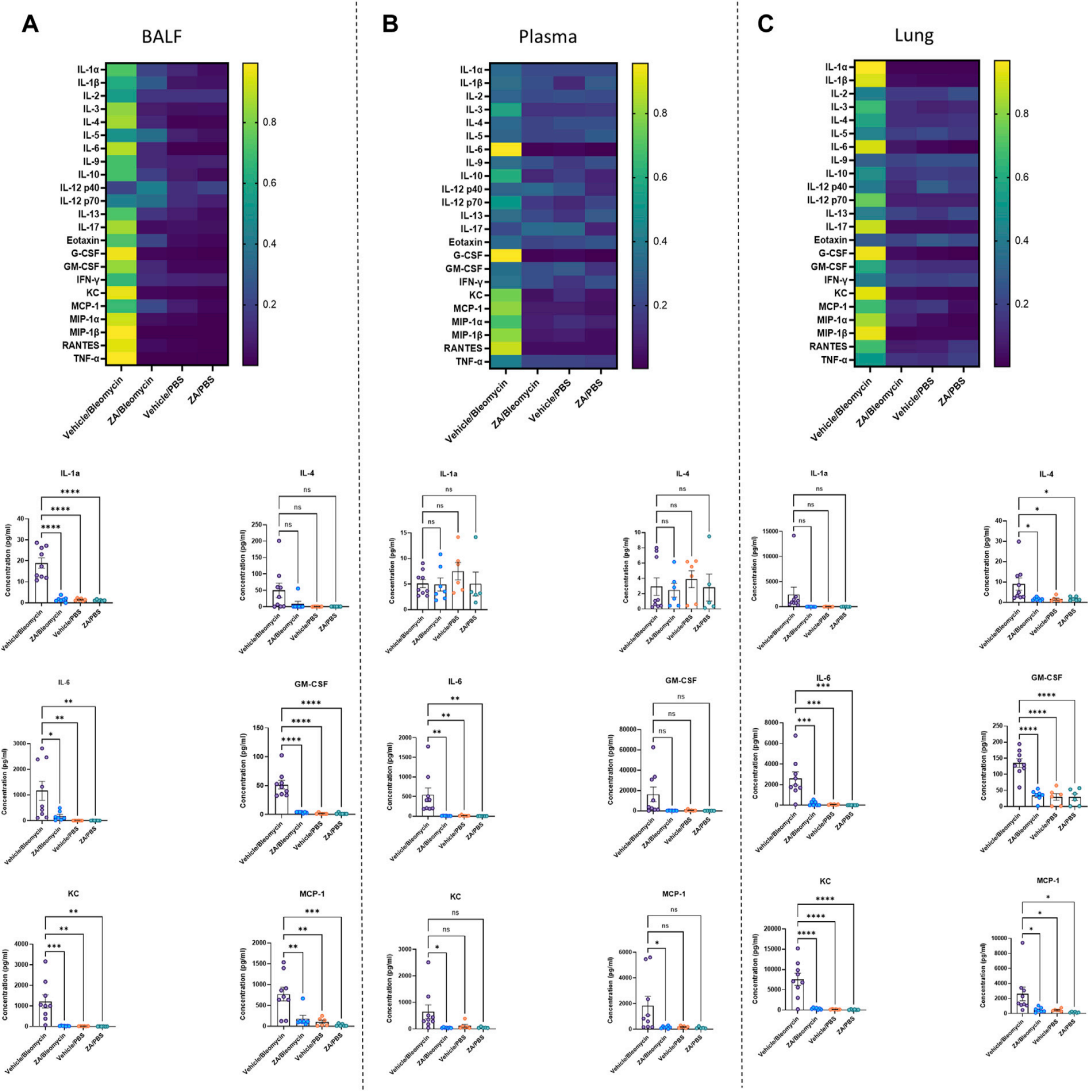
Significantly decreased murine cytokines following intraperitoneal ZA administration. Heatmaps **(A–C)** showing the differences in cytokine levels, as measured by multiplex assay, in murine BALF, plasma, and lung homogenate (yellow indicating high value; blue indicating low value) with selected corresponding significantly decreased or increased cytokine levels below. Cytokine values were compared to the vehicle/bleomycin group using a one-way ANOVA followed by a Dunnett’s post-hoc test (*p < 0.05; **p < 0.01; ***p < 0.005; ****p < 0.001).

The effect of i. p. ZA treatment on inflammatory cytokine levels was investigated in murine samples from the *in vivo* study using a 23-cytokine inflammation multiplex assay (Supplementary Figures S3–S5). The majority of cytokines were reduced in the BALF, plasma, and lung tissue following ZA treatment. In particular, lung homogenate decreases were seen for IL-4 (*p* = 0.0289), IL-6 (*p* = 0.0007), GM-CSF (*p* < 0.0001), KC (*p* < 0.0001), and MCP-1 (*p* = 0.0389) compared to the vehicle/bleomycin group (Figures 5A–C). Decreased immune cell recruitment following ZA treatment in murine BALF is supported by reduced murine cytokine levels in murine BALF and plasma. Shared decreases were seen in both fluids for IL-6, GM-CSF, KC, MCP-1, and other cytokines indicative of reduced immune cell recruitment following ZA (i.p.) treatment (Figures 5A,B; Supplementary Figure S3–S5).

### Bleomycin-Induced FDPS Production in Murine Lung Tissue Is Decreased by Zoledronic Acid Treatment

**FIGURE 6:**
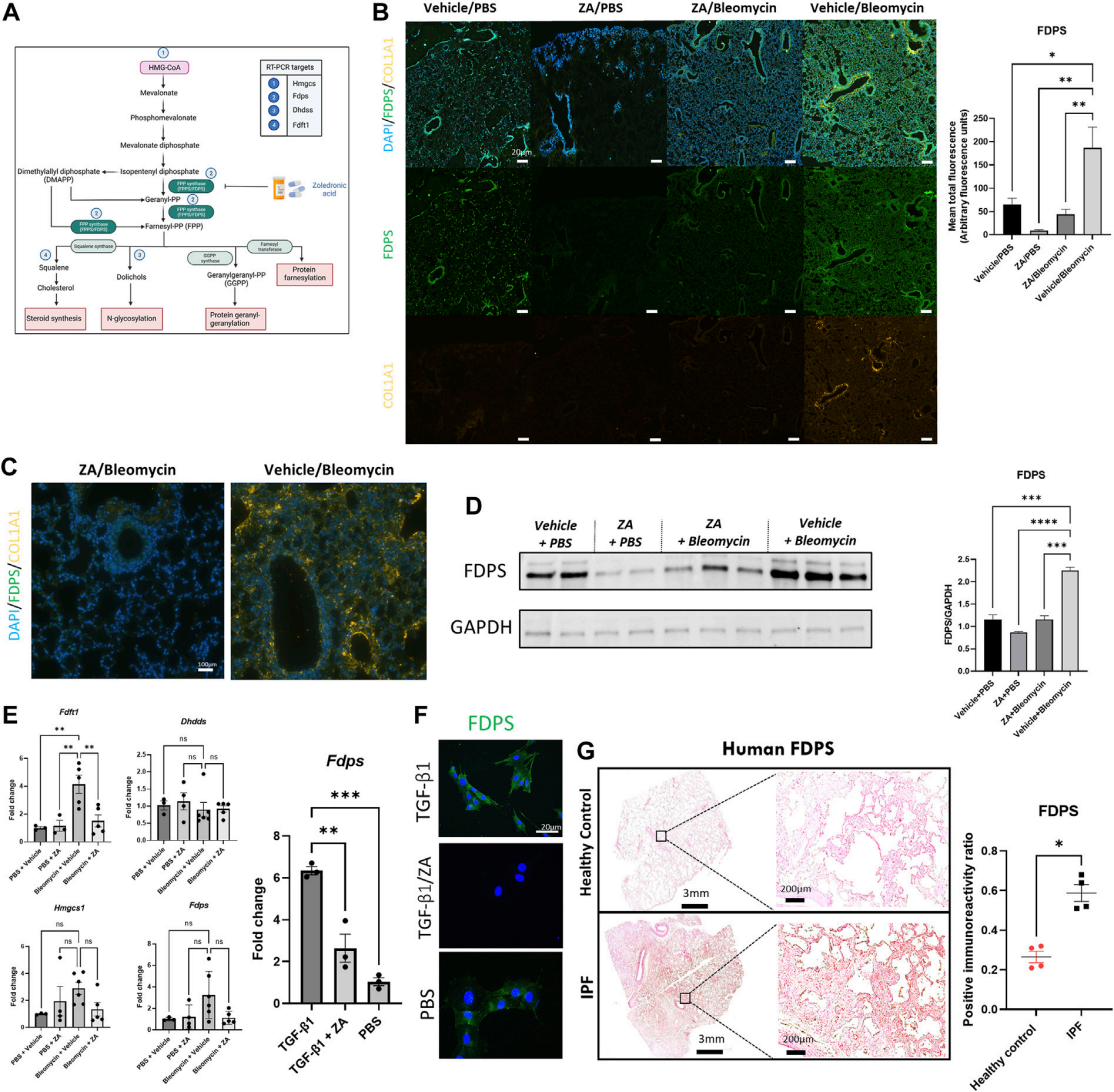
**Lower production of FDPS and COL1A1 in murine lungs upon ZA treatment. (A)** Mevalonate pathway with key enzymes and intermediates highlighted inset and numbered points indicating PCR measurements (Hmgcs: hydroxymethylglutaryl-CoA synthase; Fdps: farnesyl diphosphate synthase; Dhdds: dehydrodolichol diphosphate synthase; Fdft1: farnesyl-diphosphate farnesyl transferase). **(B)** Murine lung immunofluorescence analysis showed decreased levels of immunoreactivity for FDPS and collagen 1A1 (COL1A1) in ZA (i.p.)-treated mice compared to bleomycin (Bleo) controls (scale bar = 20 μm), with quantification of green fluorescence. ZA-treated and control samples were compared to bleomycin-treated samples using a one-way ANOVA followed by a Dunnett’s post-hoc test (*p < 0.05; **p < 0.01) and enlarged images of murine airways displaying co-staining of FDPS and COL1A1 **(C)**. **(D)** Mouse lung homogenate samples analyzed by SDS-PAGE, followed by immunoblotting using rabbit antisera specific to FDPS revealed decreased protein levels within ZA-administered mice. **(E)** RT-PCR analysis of murine lung lysates reveals decreased levels of *Fdft1* and *Fdps* in ZA-treated samples compared to bleomycin controls. Murine embryonic fibroblast cells stimulated with TGF-β1 and treated with ZA/PBS for 48 h displayed significantly reduced *Fdps* levels compared to TGF-β1 control as confirmed by **(F)** immunofluorescent staining of TGF-β1 treated MEF cells (scale bar = 20 μm). **(G)** FDPS immunoreactivity was measured in human IPF and control resected lung tissues, with IPF tissues showing significantly more immunoreactivity than control tissue (*p = 0.0286) as compared using an unpaired *t* test with Welch’s correction. Scale bar = 3 mm and 200 μm for inset images.

Increased flux through the mevalonate pathway enhances posttranslational modification of Rac1, promoting macrophage/fibroblast signaling (Larson-Casey et al., 2014, 2016; Politiek and Waterham, 2021). Further elucidation of this mechanism was conducted by probing the downstream signaling pathway (Figure 6A) by assessing farnesyl diphosphate synthase (FDPS). Bleomycin-induced FDPS production was significantly decreased following ZA administration, with ZA/bleomycin samples displaying significantly reduced fluorescence (*p =* 0.0077) and immunoreactivity compared to the vehicle/bleomycin group (Figures 6B,C). Western blot analysis revealed reduced levels of FDPS in lung homogenates in the ZA/bleomycin group compared to the vehicle/bleomycin group (Figure 6D). Furthermore, RT-PCR analysis on lung tissues showed a significantly increased expression of the downstream enzyme *Fdft1* in the vehicle/bleomycin group compared to the ZA/bleomycin group (Figure 6E). Trends towards a decreased expression of *Fdps* and the upstream enzyme *Hmgcs1*, after ZA treatment, were also found. No statistical difference in gene expression was seen for the downstream enzyme *Dhdds,* indicative of preferential targeting of the steroid synthesis pathway by ZA. RT-PCR analysis of *Fdps* expression in MEF cells after TGF-β1 exposure showed an increase in FDPS, which was reduced upon ZA treatment (Figure 6F). Immunofluorescence staining of the MEF cells revealed a similar trend with enhanced FDPS staining after exposure to TGF-β1, and a decreased staining following ZA treatment (Figure 6G).

The role of human IPF and control samples in this study was to demonstrate the potential translational utility of targeting FDPS in IPF patients. Lung samples were stained for FDPS expression using 3,3′-diaminobenzidine (DAB) chromogenic staining. Human IPF resected lung samples displayed significantly increased FDPS immunoreactivity compared to controls, indicative of a role for FDPS in IPF (Figure 6H and Supplementary Figure S6).

### Intratracheal Administration of siRNA Confirms FDPS as a Tractable Target for Idiopathic Pulmonary Fibrosis

**FIGURE 7:**
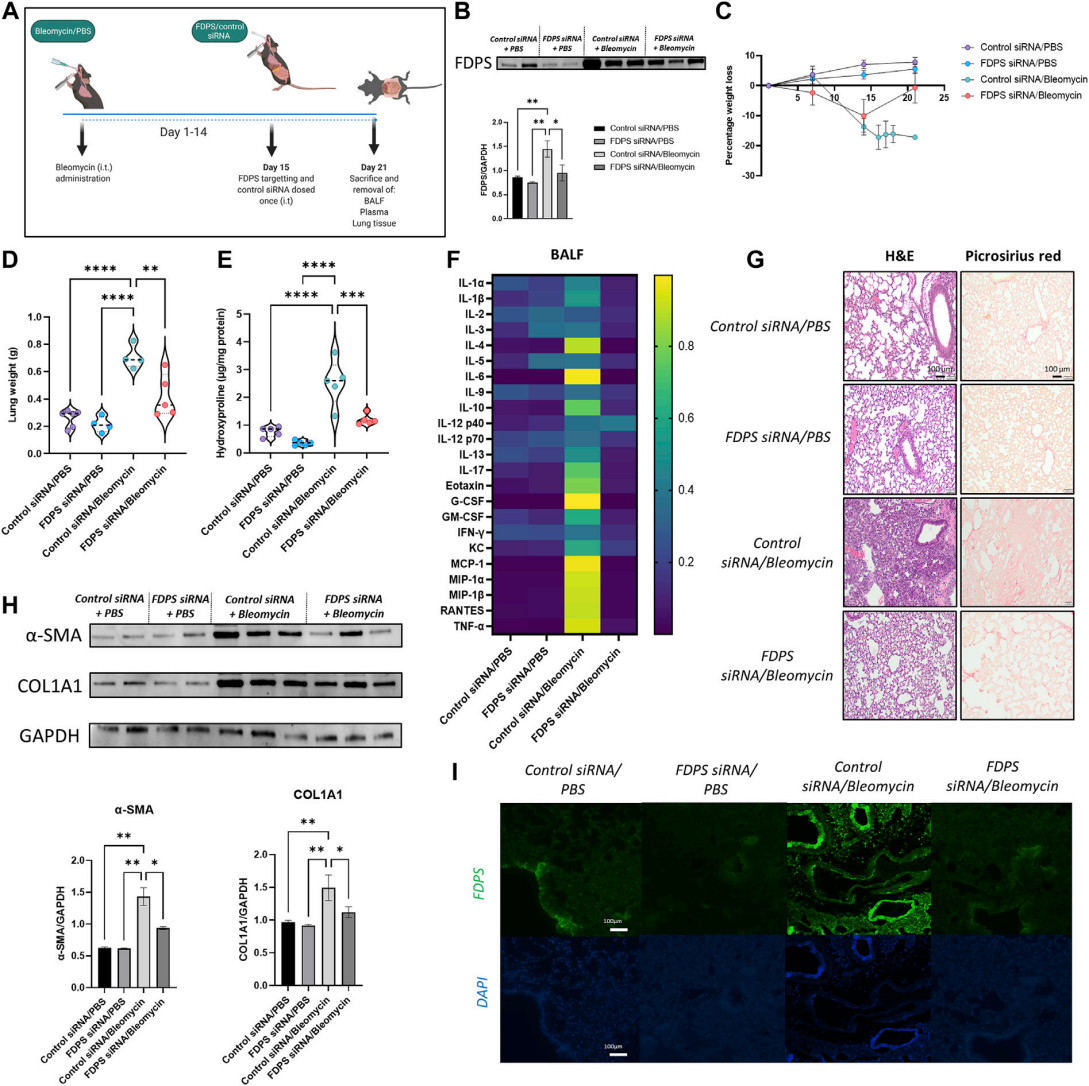
Intratracheal administration of FDPS siRNA confers protection to bleomycin-challenged mice. **(A)** Mice were subjected to bleomycin treatment at day 0, administrated FDPS or control siRNA intratracheally on day 15 and sacrificed on day 21. **(B)** SDS-PAGE followed by western blot analysis using antisera specific for FDPS in mouse lung homogenates confirms significantly decreased levels of FDPS after siRNA administration. Samples were compared using a one-way ANOVA followed by a Dunnett’s post-hoc test (*p < 0.05, **p < 0.01). **(C)** Measurement of murine weight loss following bleomycin exposure shows a protective effect of FDPS siRNA compared to control siRNA. **(D)** Bleomycin induced increases in murine lung weights were significantly reduced after FDPS siRNA treatments compared to control siRNA. Comparison between groups were performed using a one-way ANOVA followed by a Dunnett’s post-hoc test (**p < 0.01, ****p < 0.0001). **(E)** Hydroxyproline levels, which is a measurement of collagen content, was significantly increased in mice treated with bleomycin/control siRNA compared to the FDPS siRNA treated mice. Differences between groups were calculated with a one-way ANOVA followed by a Dunnett’s post-hoc test (***p < 0.001, ****p < 0.0001). **(F)** Heatmap showing the difference in inflammatory cytokine levels in BALF between treatment groups (blue indicating low value; yellow indicating high value). **(G)** Bleomycin/control siRNA mice showed increased lung damage, determined by H&E, and more intense collagen staining (picrosirius red). Lungs from mice receiving FDPS siRNA were resembling lungs from control mice regarding both H&E and collagen. **(H)** Detection and quantification of the fibrotic proteins α-smooth muscle actin (α-SMA) and collagen (COL1A1) using SDS-PAGE followed by western blot revealed a significant increase in both proteins in lungs from bleomycin/control siRNA mice, which was reduced significantly with FDPS siRNA treatment. **(I)** Immunofluorescence staining of lung sections with anti FDPS antibodies displaying an increase immunoreactivity in bleomycin/control siRNA mice, whereas lungs from mice administered FDPS siRNA were similar to lungs from control mice.

To verify that reduced FDPS levels in the lungs explains ZA’s ability to reduce bleomycin-induced lung damage, intratracheal administration of *FDPS* siRNA was used (Figure 7A). Measurement of FDPS protein levels in lung homogenates using western blot confirmed a significant reduction of FDPS levels after siRNA addition (Figure 7B). Similar to the ZA treatment, administration of *FDPS*-targeting siRNA resulted in a reduced murine weight loss (Figure 7C). Lung weights and hydroxyproline levels were significantly decreased after *FDPS* siRNA administration compared to the control siRNA/bleomycin group (Figures 7D,E). Measurements on inflammatory cytokine levels in the BALF after FDPS siRNA administration revealed a very similar pattern to the ZA treatment, whereby a reduction was seen in almost all investigated cytokines compared to the control siRNA/bleomycin group (Figure 7F and Supplementary Figure S7). Histological evaluation of lung sections from control siRNA/bleomycin treated mice revealed more closure of alveolar structures and increased collagen deposition, whereas lung sections from mice treated with *FDPS*-targeting siRNA were comparable to the control mice (Figure 7G). Furthermore, western blot analysis on synthesis of the fibrotic proteins COL1A1 and α-SMA showed a significant decrease in FDPS siRNA treated compared to control siRNA/bleomycin treated mice (Figure 7H). Finally, immunofluorescence staining of FDPS in lung sections was amplified in mice treated with control siRNA/bleomycin, which was reduced to control levels upon FDPS siRNA administration (Figure 7I).

### Farnesyl Diphosphate Synthase Targeting Results in Anti-fibrotic Macrophage Reprogramming

**FIGURE 8:**
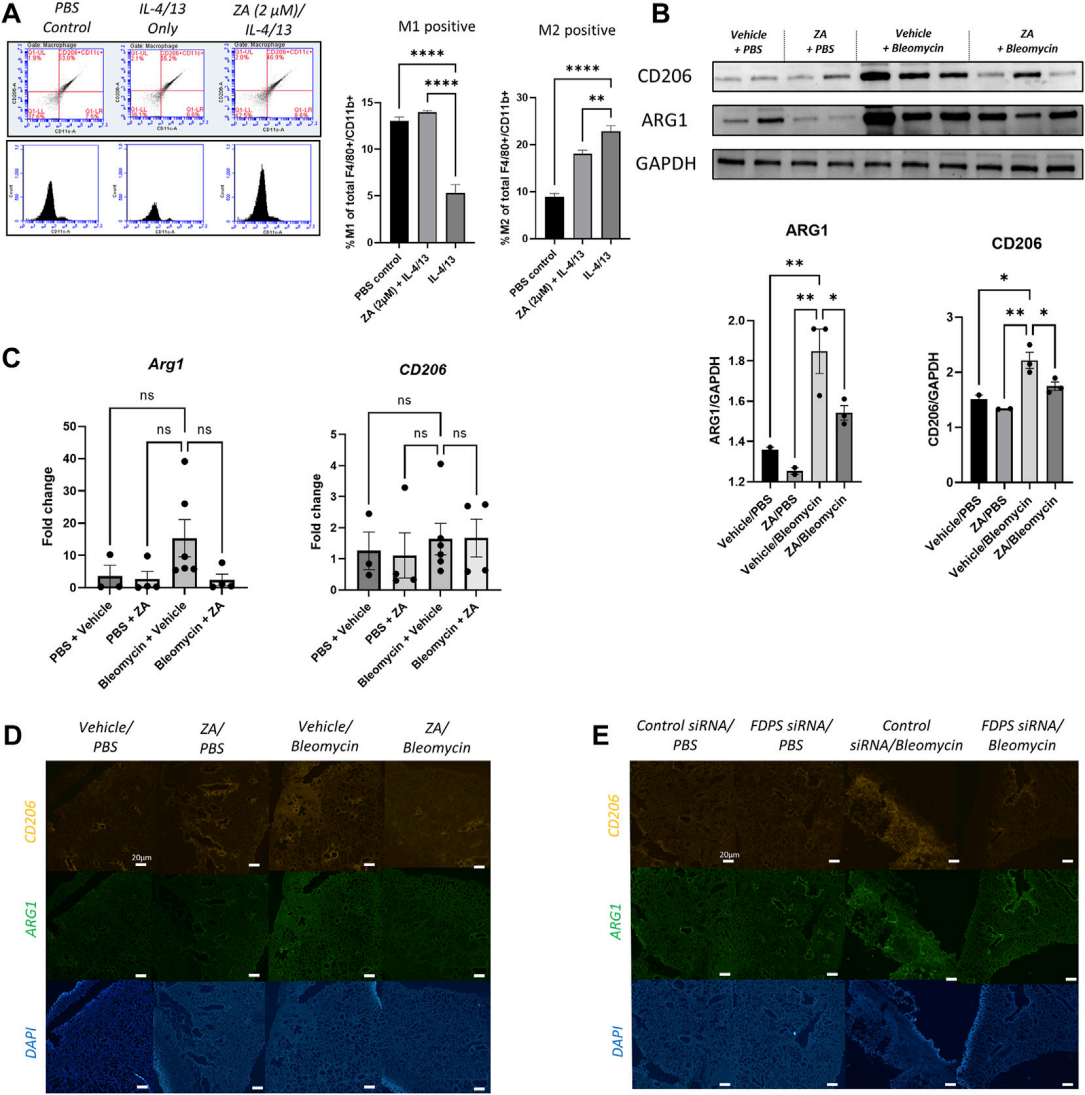
Decreased profibrotic macrophage markers following ZA or FDPS siRNA treatment **(A)** *Ex-vivo* macrophages were analyzed for M1/M2 using flow cytometry, with the gating strategy shown. Stimulation with IL-4/13 for 48 h shifted the macrophages from M1 to M2, which was in part blocked after ZA administration. M1 positive (CD206^-^/CD11c^+^/F4/80^+^/CD11b^+^) and M2 positive (CD206^+^/CD11c^−^/F4/80^+^/CD11b^+^) macrophages graphed as percentages. Statistical comparison between groups were performed using a one-way ANOVA with Dunnett’s post-hoc test (*p < 0.05; **p < 0.01; ***p < 0.001). **(B)** SDS-PAGE followed by western blot with antibodies for arginase 1 (ARG1) and cluster of differentiation 206 (CD206). Intensity quantification, normalized to Glyceraldehyde 3-phosphate dehydrogenase (GAPDH), of the bands showed decreased protein levels of ARG1 and CD206 after treatment with ZA. Statistical comparison between groups were performed using a one-way ANOVA with Dunnett’s post-hoc test (*p < 0.05; **p < 0.01; ***p < 0.001). **(C)** Real-time quantitative PCR (RT-qPCR) analysis of Arg1 and *CD206* in lung after bleomycin exposure and treatment with ZA. The mRNA expression was normalized against succinate dehydrogenase complex subunit A (*Sdha*) and fold change was calculated by difference between all treatment groups compared to PBS/vehicle. Statistical differences between groups were calculated using a one-way ANOVA with Dunnett’s post-hoc test **(D,E)** Immunofluorescence staining of ARG1 and CD206 in lung sections revealed an increased signal intensity after bleomycin exposure, which was reduced with administration of ZA **(D)** or FDPS siRNA **(E)**.

Previous studies have implicated M2 macrophages in IPF pathogenesis, with pro-fibrotic M2 macrophages characterized by expression of CD206 as well as increased metabolism of arginine into ornithine and urea by arginase (Ruaro et al., 2019; Papadimitriou et al., 2022). Stimulation of macrophages with profibrotic cytokines IL-4 and IL-13 lead to an increased number of M2 macrophages and fewer M1 macrophages indicative of a pro-fibrotic polarization. Addition of ZA to murine *ex vivo* macrophages stimulated with IL-4 and IL-13 resulted in retention of M1 macrophages and a reduction of M2 macrophages (Figure 8A). Protein levels of Arg1 and CD206 in the lung, measured by western blot, were found to be significantly increased in the bleomycin/vehicle group, whereas treatment with ZA resulted in a significant decrease of both proteins (Figure 8B). This was supported by a trend towards an increased expression of Arg1 seen in the bleomycin/vehicle animals, which was reduced after treatment with ZA (Figure 8C). No differences in expression of *CD206* were found between the groups. Immunofluorescence staining of lung sections revealed a bleomycin induced increase in fluorescence from both ARG1 and CD206 compared to the controls. The fluorescent signal for both proteins was almost undetectable in the lung sections from ZA treated mice, and a faint signal was seen in the FDPS siRNA treated mouse lungs (Figures 8D,E).

## Discussion

With current drug targets and therapies predominantly directed toward fibroblast-mediated collagen deposition and fibrotic intercession, our focus turned to indirect intervention into immune cell-mediated inhibitory activities through mevalonate intermediate targeting. The mevalonate pathway has shown utility in multiple diseases, with evidence supporting mevalonate regulation of cell proliferation and energy homeostasis (Clendening et al., 2010; Gruenbacher and Thurnher, 2017; Larson-Casey et al., 2019).

HMG-CoA reductase inhibitors such as statins, are reversible competitive inhibitors of HMG-CoA reductase, a molecule that catalyzes the de-acylation of HMG-CoA to mevalonate in cholesterol biosynthesis (Hampton et al., 1996; John R; Burnett and Vasikaran, 2002). Statins have predominantly been used to probe the mevalonate pathway in interstitial lung diseases, offering mixed results. Whilst lung function decline and hospitalizations in IPF patients from the CAPACITY and ASCEND trials was diminished by statin use (Alexeeff et al., 2007; Kreuter et al., 2017), the post-hoc analysis of the INPULSIS trials did not establish beneficial effects of statin use in IPF patients (Kreuter et al., 2018). Furthermore, an FDA report associated statin use with onset of interstitial lung disease (Fernández et al., 2008), with statins shown to increase lung fibrosis in a murine bleomycin model (Xu et al., 2012).

The approach reported in this study utilized a third-generation bisphosphonate, ZA, already employed for the treatment of osteoporosis, through potent effects against the resorptive activity of osteoclasts (Dunstan et al., 2007). This activity is mediated by inhibiting farnesyl diphosphate synthase (FDPS), an enzyme responsible for catalysing the conversion of isopentenyl pyrophosphate and dimethylallyl pyrophosphate to their geranylated and farnesylated states, respectively (Kim et al., 2018). Herein, we report several arguments supporting FDPS targeting by ZA as an appealing and clinically relevant treatment for IPF.

Reported data indicate that increased geranylgeranylation substrates increase Rac1 activity in macrophages, with resultant polarization to a profibrotic phenotype (Larson-Casey et al., 2019). Rac1 also mediates ROS generation (Osborn-Heaford et al., 2012), with IPF-derived fibroblasts and macrophages generating higher levels of ROS (Waghray et al., 2005). Furthermore, IPF-derived BAL cells displayed increased apoptotic resistance and increased TGF-β1 production (Larson-Casey et al., 2016). Our study found that TGF-β1-stimulated HFL-1 cells treated with ZA were less prone to myofibroblast transition and produced decreased levels of fibrotic proteins. RANKL induces phenotypic switching in macrophages from the initial state to M1-like cells, whereas IL-4, IL-10, IL-13, and other cytokines secreted by T helper cells are involved in M2 activation, skewing macrophages toward a profibrotic lineage (Huang et al., 2017). RANK additionally provokes biochemical signaling *via* the recruitment of intracellular TNF receptor-associated factors (TRAFs) after ligand binding and receptor oligomerization. We showed that ZA-treated monocytic cells stimulated with RANKL displayed reduced NF-κB signaling, a key regulator of proinflammatory responses in immune cells. In addition, *ex vivo* macrophage cells from murine BALF exposed to RANKL stimulation and ZA treatment displayed reduced cell recruitment efficiency.

Importantly, uncontrolled lung injury and immune cell recruitment is a hallmark of IPF initiation and progression, resulting in pro-inflammatory and pro-fibrotic cytokine release driving further fibrosis-related immune cell influx and ECM remodeling (Wynn and Ramalingam, 2012; Spagnolo et al., 2018; Upagupta et al., 2018; Baratella et al., 2021)**.** Whilst recent efforts have focused on removal of senescent or damaged fibroblasts (David et al., 2017; Schafer et al., 2017), our approach advantageously suppresses pro-fibrotic mediators and immune cell recruitment with reduced cytotoxicity.

To elucidate the mechanism by which these effects were mediated, we examined murine lung samples. Whilst no direct targeting of FDPS has, to our knowledge, been reported in the airways, FDPS has shown involvement in the progression of cardiac remodelling, with *FDPS*-targeting siRNA attenuating murine cardiac fibrosis (Zhao et al., 2016). This is further supported in our study, with *FDPS*-targeting siRNA reducing fibrotic lung damage. FDPS has displayed involvement in cardiac remodelling, with FDPS-targeting siRNA attenuating murine cardiac fibrosis (Zhao et al., 2016). Furthermore, ZA’s ability to decrease FDPS production was demonstrated in human lung tissue, murine lung tissue, and directly in fibroblast cells, correlating with subsequent decreases in collagen deposition. In addition, our study examined the most appropriate route of ZA administration, a key factor in any drug discovery program. Surprisingly, intraperitoneal administration of ZA produced the most effective alleviation of lung fibrosis. We hypothesize that intratracheal administration of ZA to the murine lung may produce cytotoxic damage or result in elimination of the macrophage population entirely [44]. Conversely, intraperitoneal introduction of ZA displayed the ability to repolarize murine macrophages to an anti-fibrotic phenotype, highlighting further advantages of ZA administration (Kaneko et al., 2018).

Important limitations addressed in this study include whether treatment using ZA can display utility in human IPF pathologies. Whilst the translational aspect of this study is suggested using murine lung sections, it is important to demonstrate that ZA treatment successfully reduces FDPS levels and subsequent IPF lung damage in human clinical trials. Transition to such clinical trials is eased by the pre-existing FDA approval of ZA (i.v.) for osteoporosis, multiple myeloma, and bone metastases in cancer.

Together, our findings demonstrate that ZA possesses a mechanism of action targeting FDPS to suppress pulmonary fibrosis, which is distinct from currently employed therapeutic interventions. This study highlights the downstream effects of FDPS inhibition, including decreasing myofibroblast transition, decreasing inflammatory cell recruitment, reducing pro-fibrotic macrophage populations, and eventual inhibition of fibrotic-related lung remodelling. These data support the use of ZA in clinical trials for the treatment of IPF.

## Data Availability

The original contributions presented in the study are included in the article/Supplementary Material, further inquiries can be directed to the corresponding author.
