# Supplementary material for: Zoledronic Acid Targeting of the Mevalonate Pathway Causes Reduced Cell Recruitment and Attenuates Pulmonary Fibrosis
Source: Front Pharmacol. 2022 Jun 2;13:899469. doi: 10.3389/fphar.2022.899469 (PMC9201219; doi:10.3389/fphar.2022.899469)
Supplement: Supplementary file 1 [file DataSheet1.docx]

Supplementary Material

Zoledronic acid targeting of the mevalonate pathway causes reduced cell recruitment and attenuates pulmonary fibrosis

[**Lloyd Tanner**](https://www.sciencedirect.com/science/article/pii/S156919932030802X#!)**^1*^, Jesper Bergwik^1^, Andrew B. Single^1^†, Ravi KV Bhongir^1^, Jonas S. Erjefält^2^, Arne Egesten^1^**

^1^Respiratory Medicine, Allergology, and Palliative Medicine, Department of Clinical Sciences Lund, Lund University and Skåne University Hospital, Lund, Sweden

^2^Unit of Airway Inflammation, Department of Experimental Medical Sciences, Lund University, Lund, Sweden

† Present address: Norwegian University of Science and Technology (NTNU), Trondheim, Norway

*** Correspondence:**Lloyd Tanner ([lloyd.tanner@med.lu.se](mailto:lloyd.tanner@med.lu.se))

Keywords: IPF, zoledronic acid, mevalonate, drug-repurposing, fibrosis, FDPS

**Supplemental Figures**

**
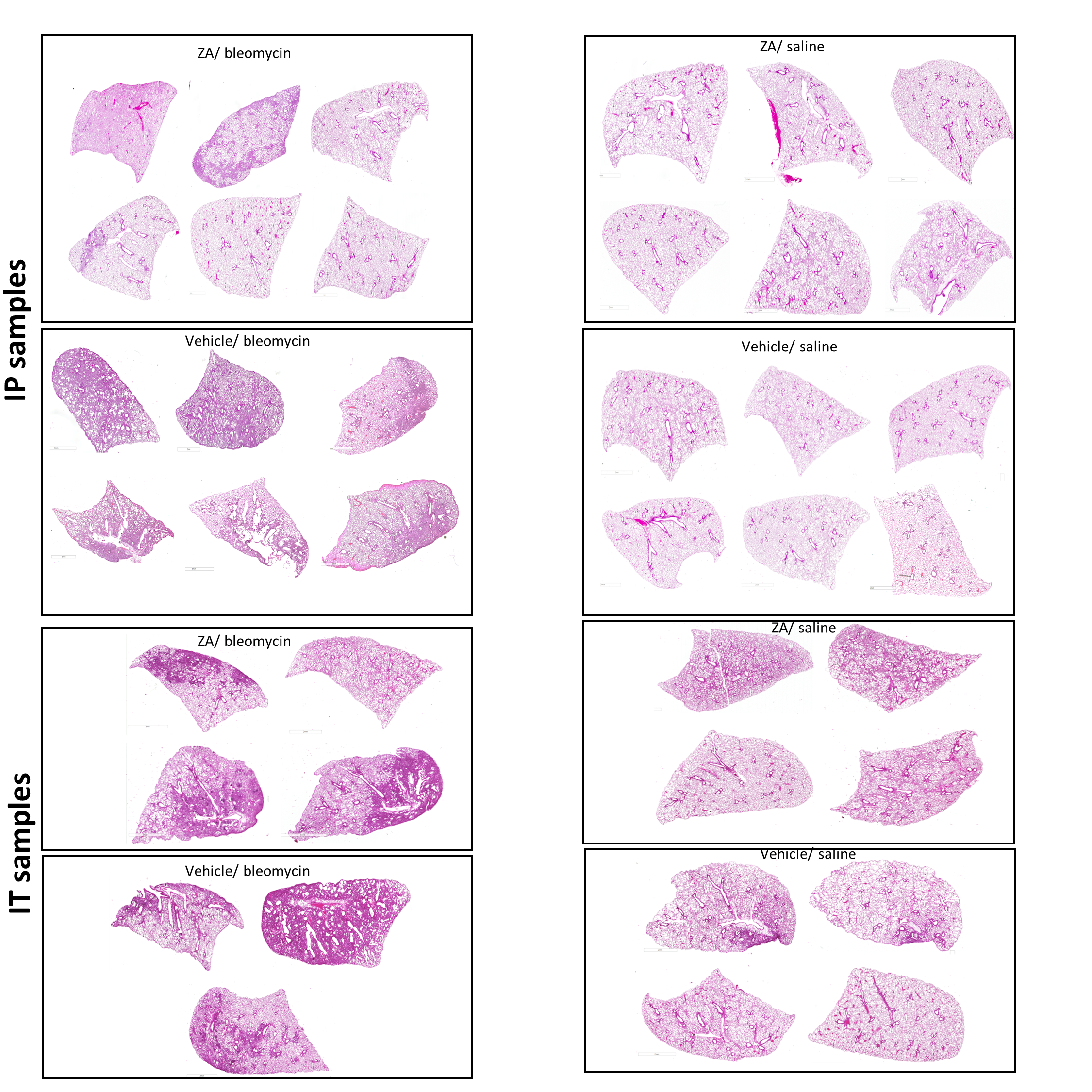
**

**Supplementary Figure 1: Whole lung scans of murine lungs from ZA experiments following H&E staining (scale bar=2 mm).**

**
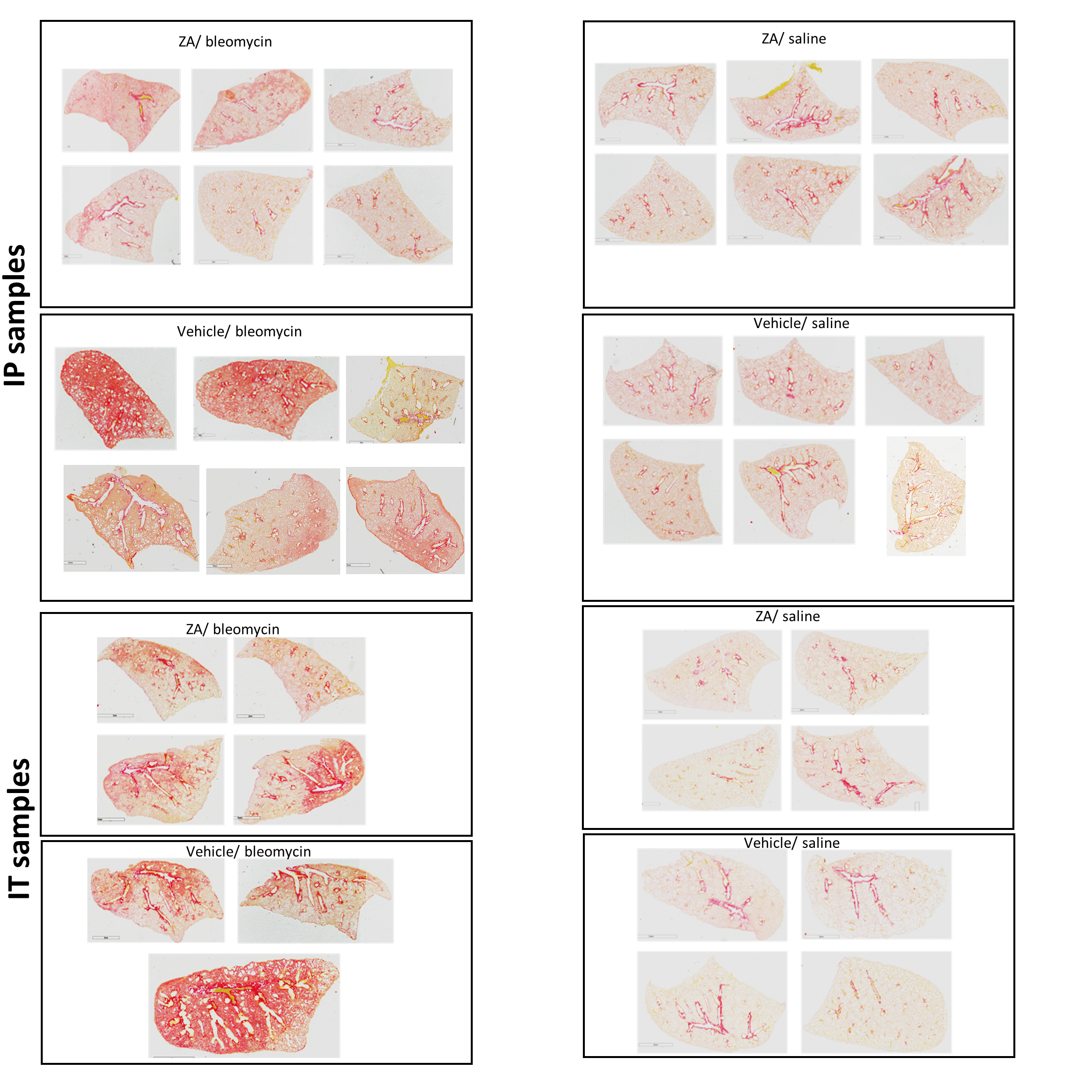
**

**Supplementary Figure 2: Whole lung scans of murine lungs from ZA experiments following picrosirius red staining (scale bar=2 mm).**

**
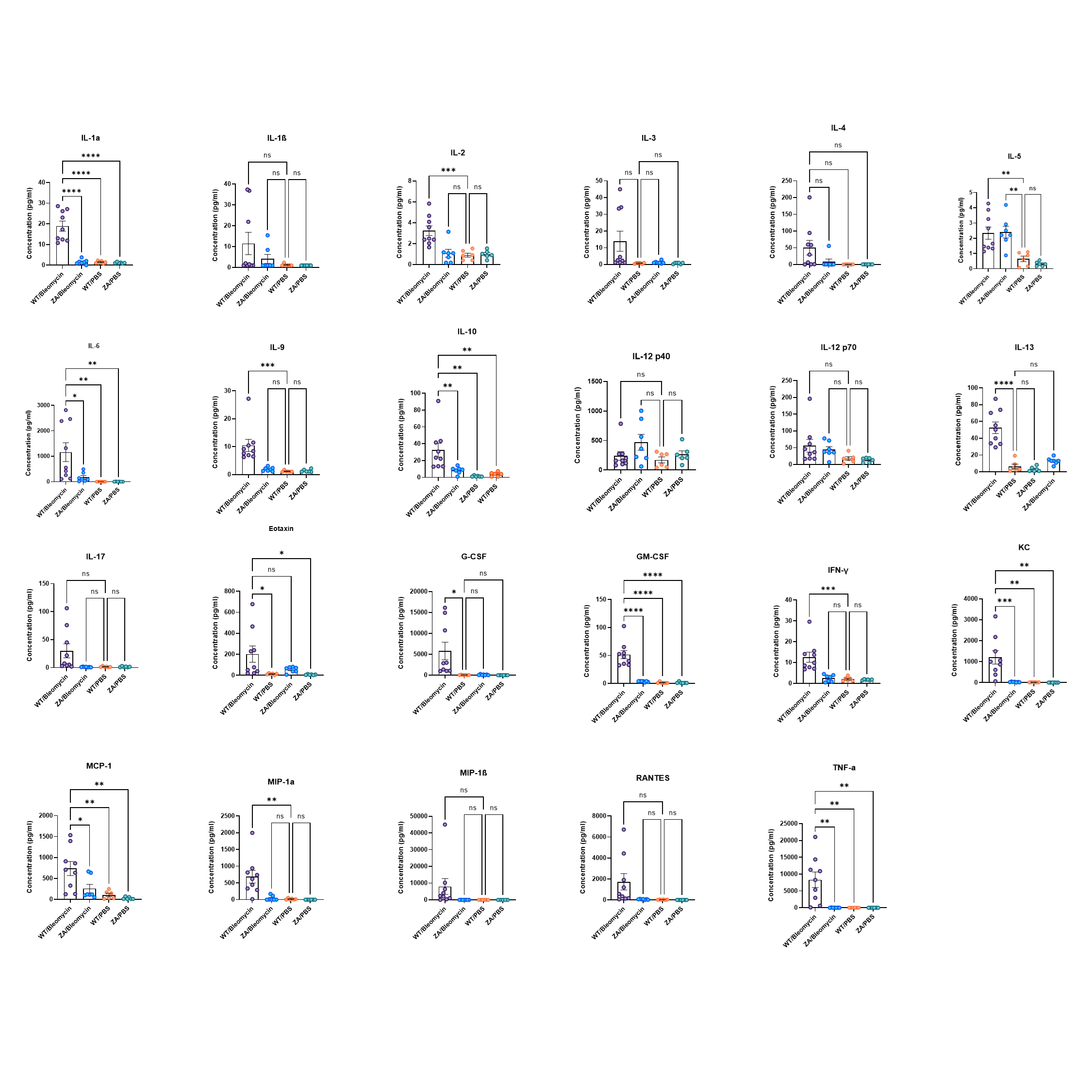
**

**Supplementary Figure 3: Murine BALF cytokine levels from ZA (i.p.) experiments.** Cytokine values were compared to the vehicle/bleomycin group using a one-way ANOVA (**P<*0.05*; **P<*0.01*; ***P<*0.005*; *****P<*0.001).

**
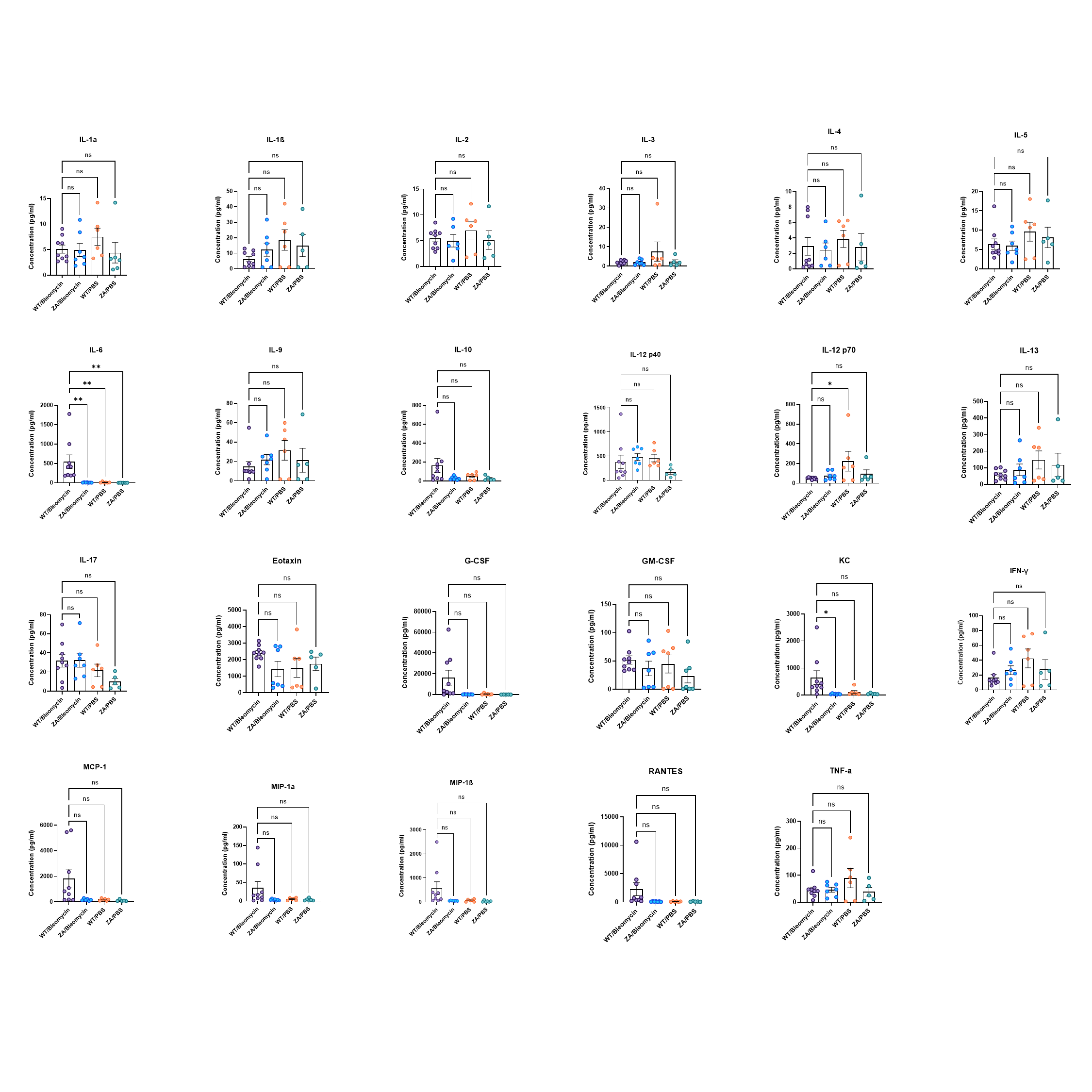
**

**Supplementary Figure 4: Murine plasma cytokine levels from ZA (i.p.) experiments.** Cytokine values were compared to the vehicle/bleomycin group using a one-way ANOVA (**P<*0.05*; **P<*0.01*; ***P<*0.005*; *****P<*0.001).

**
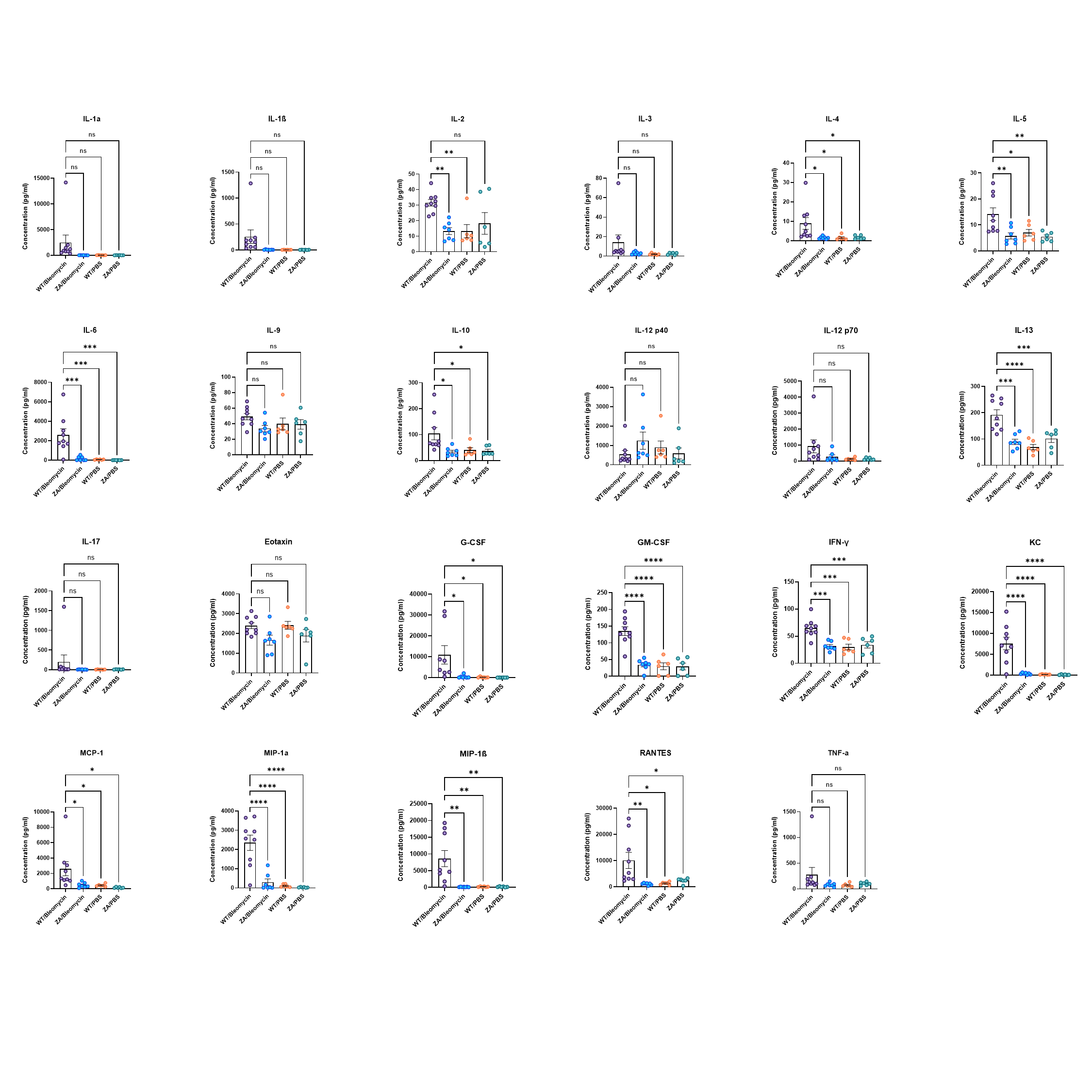
**

**Supplementary Figure 5: Murine lung homogenate cytokine levels from ZA (i.p.) experiments.** Cytokine values were compared to the vehicle/bleomycin group using a one-way ANOVA (**P<*0.05*; **P<*0.01*; ***P<*0.005*; *****P<*0.001).


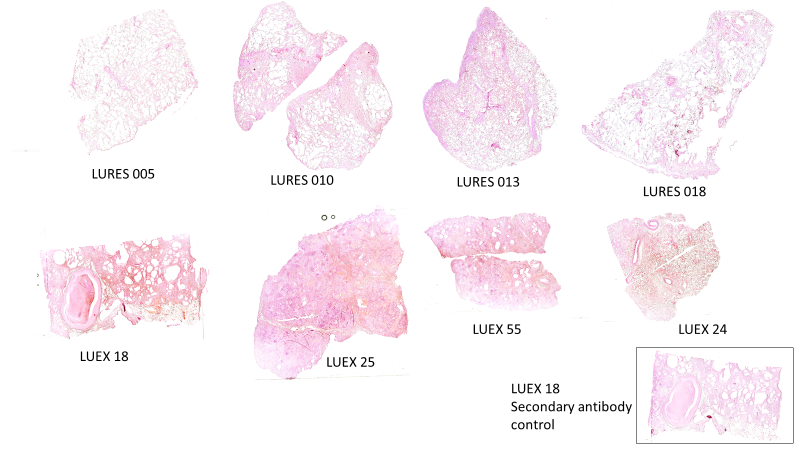


3mm

**Supplementary Figure 6:** Lung scans of human lung explants following immunostaining for FDPS (scale bar=3 mm). Inset, antibody staining control containing only secondary antibody.

**Supplementary Figure 7: Murine BALF cytokine levels from siRNA experiments.** Cytokine values were compared to the vehicle/bleomycin group using a one-way ANOVA (**P<*0.05*; **P<*0.01*; ***P<*0.005*; *****P<*0.001).


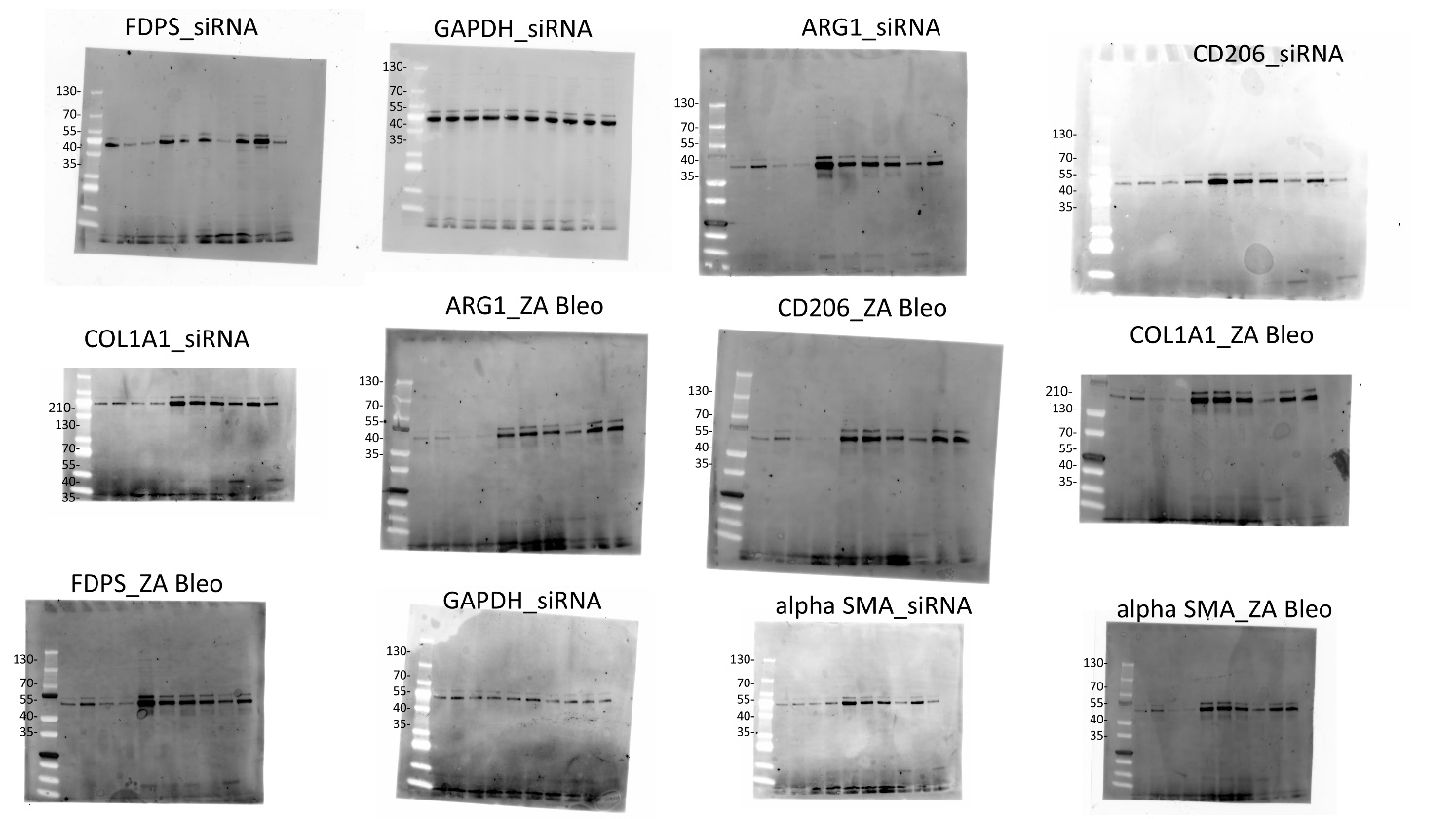


**Supplementary Figure 8: Representative Western blots conducted on murine lung tissue in ZA/bleomycin and FDPS siRNA experiments.** GAPDH was utilized as a control for all murine lung sample blots.

**Supplementary Table 1: List of probes used for Real-time PCR analysis.**

| **Gene** | **Probe** |
| --- | --- |
| *Sdha* | Mm01352366_m1 |
| *Fdft1* | Mm01598574_g1 |
| *Fdps* | Mm00836315_g1 |
| *Dhdds* | Mm00482843_m1 |
| *Hmgcs1* | Mm01304569_m1 |
| *Arg1* | Mm00475988_m1 |
| *Cd206* | Mm01329359_m1 |

**Supplementary Table 2: Showing clinical data for patient samples included in this study. Healthy control samples were obtained from cancer patients undergoing lung resectioning (values not available-NA; F-female; M-male).** FEV1- Forced expiratory volume in one second; FEV1%-percentage forced expiratory volume % of expected FEV1 (in relation to age, height, and sex); FEV1/FVC- represents the proportion of a person's [vital capacity](https://en.wikipedia.org/wiki/Vital_capacity) expired in the first second of forced expiration ([FEV1](https://en.wikipedia.org/wiki/Spirometry#Forced_expiratory_volume_in_1_second_(FEV1))) divided by the forced vital capacity (FVC); DLCO-diffusion capacity of the lungs to carbon monoxide (CO per unit time per mm of driving pressure of CO (cc of CO/sec/mm of Hg)).

| **Patient ID** | **Sex** | **Diagnosis** | **FEV1** | **FEV1%** | **FVC** | **FEV1/FVC** | **DCLO (%)** |
| --- | --- | --- | --- | --- | --- | --- | --- |
| LURES 005 | F | Adenocarcinoma | 2.0 | 90 | 2.46 | 0.81 | 50 |
| LURES 010 | F | Atypical carcinoma | 1.78 | 59 | 2.37 | 0.75 | 64 |
| LURES 013 | F | Adenocarcinoma | 2.5 | 101 | 3.37 | 0.74 | 96 |
| LURES 018 | M | Squamous epithelial cell cancer | 3.1 | 93 | NA | NA | NA |
| LUEX 18 | M | Lung Fibrosis | 2.2 | 53 | 2.78 | 0.79 | 34 |
| LUEX 25 | M | Lung Fibrosis | 1.22 | 41 | 1.56 | 0.81 | NA |
| LUEX 55 | M | Lung Fibrosis | 2.2 | 73 | 2.52 | 0.87 | 26 |
| LUEX 24 | F | Lung Fibrosis | 1.32 | 53 | 1.76 | 0.75 | 33 |
